# Significant abundance of *cis* configurations of coding variants in diploid human genomes

**DOI:** 10.1093/nar/gkz031

**Published:** 2019-01-30

**Authors:** Margret R Hoehe, Ralf Herwig, Qing Mao, Brock A Peters, Radoje Drmanac, George M Church, Thomas Huebsch

**Affiliations:** 1 Department of Computational Molecular Biology, Max Planck Institute for Molecular Genetics, 14195 Berlin, Germany; 2 Complete Genomics, Inc., San Jose, CA 95112, USA; 3 BGI-Shenzhen, Shenzhen 518083, China; 4 Department of Genetics, Harvard Medical School, Boston, MA 02115, USA

## Abstract

To fully understand human genetic variation and its functional consequences, the specific distribution of variants between the two chromosomal homologues of genes must be known. The ‘phase’ of variants can significantly impact gene function and phenotype. To assess patterns of phase at large scale, we have analyzed 18 121 autosomal genes in 1092 statistically phased genomes from the 1000 Genomes Project and 184 experimentally phased genomes from the Personal Genome Project. Here we show that genes with *cis*-configurations of coding variants are more frequent than genes with *trans*-configurations in a genome, with global *cis/trans* ratios of ∼60:40. Significant *cis*-abundance was observed in virtually all genomes in all populations. Moreover, we identified a large group of genes exhibiting *cis*-configurations of protein-changing variants in excess, so-called ‘*cis*-abundant genes’, and a smaller group of ‘*trans*-abundant genes’. These two gene categories were functionally distinguishable, and exhibited strikingly different distributional patterns of protein-changing variants. Underlying these phenomena was a shared set of phase-sensitive genes of importance for adaptation and evolution. This work establishes common patterns of phase as key characteristics of diploid human exomes and provides evidence for their functional significance, highlighting the importance of phase for the interpretation of protein-coding genetic variation and gene function.

## INTRODUCTION

The analysis of human genetic variation has focused so far on the identification, cataloguing and annotation of variants, particularly in protein-coding genes (1,2). One key aspect of genetic variation has, however, not yet been fully addressed: the distribution of variants between the two homologous chromosomes in a diploid genome. Whether variants reside on the same homologue, in *cis*, or on both homologues, in *trans*, is ‘key to understanding their impact on gene function and phenotype’ (3,4). For example, while two null mutations in *cis* leave the second form of the gene intact, no functional form of the gene is present in a *trans* configuration, changing the phenotype entirely (5). Thus, to fully understand the biology of genes and genomes, the phase of functionally relevant variants must be known. In a first preliminary population level analysis of haplotype-resolved genomes (6), we have recently shown a non-random distribution of protein-changing variants between homologous gene forms, as indicated by significant abundance of *cis* configurations with an average *cis/trans* ratio of approximately 60:40. This raises the following questions: Could *cis* abundance of protein-changing variants, that is their preferential location on the same chromosomal homologue of an autosomal gene, represent a universal characteristic of diploid human genomes? And if so, what are the potential biological implications of this phenomenon? Thus, we aimed to (i) assess this phenomenon in substantially larger numbers of genomes and different populations, (ii) identify the genes underlying this phenomenon, (iii) test these genes for significant excess of either *cis*, or *trans* configurations across the genomes under study and (iv) functionally characterize the genes found to differ in ‘configuration type’.

We assessed the *cis/trans* ratios of protein-coding variants in 1092 statistically haplotype-resolved genomes from the 1000 Genomes database (1), and each of the different ancestry groups, and populations, contained therein separately. The key results obtained from this resource were then cross-validated in an independent sample of 184 experimentally phased genomes from the Personal Genome Project (PGP) (7,8), a huge advance over our earlier analysis of 14 molecularly haplotype-resolved genomes (6). *Cis/trans* ratios were assessed for population samples as a whole as well as for each individual genome separately as described in more detail in the Methods section, which also provides further information on the concepts and definitions underlying our approach. The ratios were determined for non-synonymous single nucleotide polymorphisms (nsSNPs) predicted to alter protein function, for the entirety of nsSNPs, for all synonymous SNPs (sSNPs), and for combinations of these types of coding variants as they co-occur within the genes. Once significant abundance of *cis* configurations was obtained, *cis/trans* ratios were further dissected in genetic and genomic context. To address the potential functional implications of this phenomenon, we focused on the genes exhibiting ≥2 nsSNPs predicted to affect protein function in either *cis*, or *trans* configurations. The fractions of these genes being highly similar between the genomes, we verified existence of a shared, global set of ‘phase-sensitive’ genes with ≥2 protein function-altering nsSNPs. Subsequently scoring *cis* and *trans* configurations per gene unveiled a larger group of *cis*- and smaller group of *trans*-abundant genes, resulting in observed global *cis/trans* ratio of ∼60:40 as the net effect. These two categories of autosomal protein-coding genes were further functionally differentiated by gene ontology (GO) and pathway analysis as well as characterized by different distributional patterns of protein function-altering variants with potential implications for protein structure. Thus, the distinction of *cis* and *trans* configurations ultimately leads to the classification of variable autosomal genes into two major, potentially functionally divergent, categories. This work provides a first phase-informed global view of the diploid human exome in aggregate and across multiple populations, highlighting the importance of phase for the interpretation of protein-coding genetic variation, with implications for the conceptual and functional characterization of autosomal genes in the context of a diploid biology.

## MATERIALS AND METHODS

### 1000 Genomes (1000G) Consortium haplotype data

Haplotype data from 1092 genomes (1) were downloaded from ftp.1000genomes.ebi.ac.uk/vol1/ftp/phase1/analysis_results/shapeit2_phased_haplotypes/. *Cis* and *trans* configurations were assessed in the total of 1092 genomes and separately in the four ancestry groups (EUR, European, *n* = 379; EAS, East Asian, *n* = 286; AMR, Americas, *n* = 181; AFR, African, *n* = 246) and 14 populations contained therein. Phased data were available across all 1092 genomes, with ‘no call’ rates between 2.1 and 6% and routine use of imputation in the case of missing data. Regarding the accuracy of inferred haplotypes, a phasing switch error every 300–400 kb on average has been estimated (1). Notably, exome data had a very high coverage due to the integration of additional, targeted deep (50–100×) exome sequence and dense SNP genotype data (details in (1)).

### 184 experimentally phased genomes from the Personal Genome Project (PGP) database

Individuals were recruited as part of PGP (7,8). Participants enrolled in the PGP gave full consent to have their genotypic and phenotypic data made freely and publically available. Documents reviewed and signed by each participant can be found at http://www.personalgenomes.org/harvard/sign-up. Each participant provided a blood sample, self-reported ethnicity, and detailed phenotype information. High molecular weight DNA was isolated, as previously described (9), for haplotype-resolved whole genome analysis using Complete Genomics’ Long Fragment Read technology (9). This enabled > 98% of all heterozygous SNPs to be placed into contigs with an average N50 of 800 kb (7). Importantly, a large number of technical replicate samples were generated to measure phasing error rates. As also demonstrated previously (9), these error rates were exceedingly low with average short and long switch error rates (SERs) of 0.00068 and 0.00051, respectively (for comparison see the higher SERs reported for other phasing approaches by Choi *et al.* (10)). This enabled ∼86% of overlapping blocks between replicate samples to be completely error-free (7). Importantly, for analyses in this study, a contig filter was applied to ensure that phase configurations were determined only for those coding variants that were contained within the same contig.

### Approaches, concepts and definitions

Where diploid autosomal genes have ≥2 heterozygous variants, these can either reside on the same chromosomal homologue, in *cis*, or on both homologues, in *trans* (Supplementary Scheme S1, Supplementary Figure S1A). ‘Phase’ is always determined for nucleotides/alleles different from the reference sequence, also defined as ‘non-reference alleles’. The non-reference allele is in the vast majority, but not all cases, the minor allele. Genes with ≥2 heterozygous variants which could exist in either phase configuration are defined as ‘phase-sensitive’. The distinction of *cis* and *trans* configurations is based on haplotype information and precisely refers to the *pair* of haplotypes corresponding to the two parental homologues of a specific autosomal protein-coding gene (primary transcript) in an individual genome. This needs to be distinguished from the ‘haplotypes’ generated by the HapMap and 1000 Genomes Projects. These projects have established a ‘map’ of the unique, common haplotypes underlying genetic variation in populations to employ linkage disequilibrium (LD) measures aimed at inferring unobserved (disease) variants. By contrast, our approach to the analysis of haplotypes focuses on the potential functional significance of phase. Thus, the pairs of haplotypes examined correspond to the protein-coding regions of the genes and as such are confined functional units where differences in the phase of non-synonymous variants may have functional consequences. For example, nsSNPs that reside in *cis* leave a second form of the gene unperturbed, while nsSNPs in *trans* may affect both homologous forms of the gene.

Phase is most likely to impact gene function and phenotype within genes that contain nsSNPs of potential functional significance. The annotation of nsSNPs is achieved by use of algorithms (below) which predict whether missense changes caused by nsSNPs alter protein function. Thus, in the first part of the analysis, which focuses on the comprehensive evaluation of *cis/trans* ratios, the phase configurations were initially assessed for such predicted protein function-altering nsSNPs (PFA-nsSNPs). Additional analysis of all nsSNPs and all sSNPs, respectively, should clarify if only the potentially functionally significant PFA-nsSNPs show *cis* abundance, or all three classes of coding variants together. In the first case, this could indicate selection, in the second case a common underlying mechanism, most likely LD. Finally, the *cis/trans* ratios were assessed using all these types of coding variants as they co-exist within the genes. In the second part of the analysis, we focused primarily on the genes containing ≥2 PFA-nsSNPs to examine the potential functional implications of significant *cis* abundance.

Any result concerning *cis/trans* ratios in this work was based on distinct analysis of individual genomes. The *cis/trans* ratio of an individual genome was determined as follows: each autosomal protein-coding gene with ≥2 PFA-nsSNPs was assigned a *cis* or *trans* configuration. This allowed immediate calculation of the *cis* fraction (%) of an individual genome as the number of autosomal genes with *cis* configurations divided by the total number of genes with ≥2 PFA-nsSNPs, i.e. total configuration count (equivalent to 100%), and of the *trans* fraction (%) per genome as 100%–*cis* (%). Thus, the *cis/trans* ratio of an individual genome represents the ratio of *cis* fraction to *trans* fraction. To determine the *cis/trans* ratios for totals of 1092 or 184 genomes, or any subsets of these, the median values of the *cis*, and *trans* fractions obtained across defined numbers of genomes were calculated. As indicated, *cis/trans* ratios were determined for PFA-nsSNPs in a first pass, and for the other classes of coding variants (separately) in a second pass. Because these *cis/trans* ratios result from the analysis of whole genomes, they are also termed ‘global’ *cis/trans* ratios.

As outlined above, the evaluation of *cis* and *trans* configurations is based on a *pair* of haplotypes that correspond to a diploid gene within an individual haplotype-resolved genome. Current approaches do not allow, however, distinction of maternal and paternal haplotypes, but solely the distinction of two different combinations of heterozygous variants. These are designated ‘Haplotype 1’ and ‘Haplotype 2’. Supplementary Scheme S2 (Supplementary Figure S1B) provides an overview of all different pairs of maternal and paternal haplotypes expected to occur in a population if a defined number of heterozygous variants (2 and 3, respectively, in this example) in a protein-coding gene are distributed randomly between the parental homologues. This scheme illustrates, firstly, that the mere distinction of two different haplotypes without knowledge of parental origin does not affect the *cis* and *trans* fractions, i.e. the *cis/trans* ratios obtained, and secondly, that the number of *cis* configurations (2) remains constant with increasing numbers of heterozygous variants in a gene, while the number of *trans* configurations grows exponentially under random assumptions (see also graph, Supplementary Figure S1B). This graph, moreover, provides a comparative evaluation of expected versus observed *cis/trans* ratios.

### Annotation of coding variants

RefSeq genes were downloaded from UCSC table browser (Hg19). All transcripts belonging to an autosomal gene were merged and the coordinates defining the entire gene region determined, resulting in a final set of 18 121 autosomal protein-coding genes. The proportion of pseudogenes (‘processed’ and ‘unprocessed’ pseudogenes as identified with BioMart) in these data is 0.7% and as such does not interfere with subsequent genome-wide statistics. For analysis of the 1092 genomes, the annotation of nsSNPs and sSNPs, and of PFA-nsSNPs, respectively, were provided by the 1000G database. The annotation of nsSNPs and sSNPs in PGP data were provided by the PGP database (7). To predict the effect of nsSNPs on protein structure and function in PGP, a combination of PolyPhen-2 (11) and SIFT (12) (see also (6)) as well as GERP conservation scores (13) were applied to ensure comparability with the 1000G annotations. Default threshold values for PolyPhen-2 and SIFT, and GERP scores > 2, were used.

### Assessment of *cis* and *trans* configurations

Each of the 1092 genomes was filtered for genes containing PFA-nsSNPs and 1092 intermediate data output files were prepared. These were organized in table format as follows: ‘Haplotype 1’ and ‘Haplotype 2’, together representing an individual (diploid) genome, were contained in two adjacent columns. The rows specified the heterozygous genomic coordinates and their gene IDs. (Homozygous variants were excluded from analysis.) Within each row, the two adjacent cells within columns ‘Haplotype 1’ and ‘Haplotype 2’ contained the two alleles at the specified heterozygous positions in the gene; one of the cells contained the reference allele designated ‘0’, and the other the non-reference allele designated ‘1’. Thus, each of the two columns/haplotypes was characterized by a unique combination of ‘1's and ‘0's. Both haplotype columns consist of as many rows per gene ID as there are heterozygous PFA-nsSNPs in the gene. In this context it seems important to note that, even though a specific gene may have multiple heterozygous sites in a population, its number of heterozygous positions in an individual genome is limited to very few. For the analysis of PGP data, PolyPhen-2 and SIFT in combination with GERP (as described) were applied to the PGP output files generated from each of the 184 experimentally phased genomes, and the intermediate output files were prepared accordingly.

In the next step, all genes with only one row corresponding to a gene ID, i.e. with only one PFA-nsSNP, were removed from the intermediate output files to ensure that only genes with ≥2 such PFA-nsSNPs were included. Then the alleles which were assigned to the same gene ID and sorted 5′ to 3′ within both ‘Haplotype’ columns, were stored as units. These units were then subjected to the assessment of phase configurations. Notably, while it is a *pair* of haplotypes that underlies a *cis* or *trans* configuration, it is sufficient to inspect only *one* haplotype, per definition ‘Haplotype 1’, to be able to score a gene *‘cis’* or *‘trans’*. Thus, a gene is classified *‘cis’* if every allele is either 1 or 0 (non-reference or reference), otherwise *‘trans’*. Thus, for each genome, a result file was generated which contained the gene IDs with an assignment of *cis* or *trans*. This allowed immediate calculation of the global *cis/trans* ratios as described above (see also (6)). The *cis/trans* ratios for the other classes of coding variants were assessed analogously. All bioinformatic steps involved were automated for large scale analysis.

To evaluate the significance of a given *cis/trans* ratio in an individual genome, we derived the composite probability of a *cis*, or *trans* configuration across all genes. We can model the probability of an observed *cis*, or *trans* configuration in a gene with *i* variants with a Bernoulli experiment ${P_i}( {X\ = \ 1} )$ where $X\ = \ 1$ denotes a *cis* configuration and $X\ = \ 0$ a *trans* configuration. Thus, we have ${P_i}\ ( {X\ = \ 1} ) = \frac{1}{{{2^{i - 1}}}}$ and ${P_i}\ ( {X\ = \ 0} ) = \ 1 - \frac{1}{{{2^{i - 1}}}}$ taking into account the genomic order of the variants. Among the number of genes with ≥2 variants that have either *cis* or *trans* configurations, let ${w_i}$ be the relative frequency of genes with exactly *i* variants. Thus, we have $\mathop \sum \nolimits_{i \ge 2} {w_i} = \ 1.$ The probability of observing a *cis* configuration among all phase-sensitive genes in a genome is then given by the weighted sum of the above defined Bernoulli probabilities: $P\ ( {X\ = \ 1} ) = \mathop \sum \nolimits_{i \ge 2} {w_i}\ {P_i}( {X\ = \ 1} )$. Thus, inserting the observed relative frequencies, ${w_i}$, yielded an expected probability of ∼0.4 for a *cis* configuration to occur (Supplementary Table S2A). The significance of the observed *cis/trans* ratio for a given genome was then computed with an exact Binomial test with *P* = 0.4. Notably, this probability can also be derived by simulation of phased genomes (Supplementary Results andSupplementary Table S3; Supplementary Methods). To assess the significance values for a *cis/trans* ratio which was calculated for a defined population sample, such as the 1092 or 184 genomes, we derived the median values for both *cis* and *trans* configurations across all genomes. This ‘median genome’ was then treated as an individual genome as described above. Thus, the significance values estimated for global *cis/trans* ratios most likely represent an underestimation.

### Distinction of *cis*- and *trans*-abundant genes

To identify *cis-* or *trans*-abundant genes, that is, genes with ≥2 PFA-nsSNPs exhibiting either configuration in significant excess, we assessed ‘gene-based’ *cis* or *trans* fractions, respectively. These were calculated as the number of *cis*, or *trans* configurations observed for a gene across all genomes in a defined population sample, divided by the total configuration count (100%) of this gene in the sample. Significant abundance of either configuration was evaluated with a Binomial test (*P* < 0.05).

### Over-representation analysis

In order to assess significance of over-representation of gene lists in pre-annotated gene sets (for example pathways, GO terms, or pre-defined gene lists) we used the hyper-geometric distribution. For each annotation set, the *P*-value is calculated as

\begin{equation*}P{\rm{\ }}\left( {x{\rm{|}}n,m,N} \right) = {\rm{\ }}1 - \mathop \sum \nolimits_{i{\rm{\ }} = {\rm{\ }}0}^{x - 1} \frac{{\left( {\begin{array}{@{}*{1}{c}@{}} m\\ i \end{array}} \right)\left( {\begin{array}{@{}*{1}{c}@{}} {N - m}\\ {n - i} \end{array}} \right)}}{{\left( {\begin{array}{@{}*{1}{c}@{}} N\\ n \end{array}} \right)}},\end{equation*}

where *x* is the number of entities in the respective gene list that overlap with the entities in the annotation set, *n* is the total number of entities in the annotation set, *m* is the total number of entities in the gene list and *N* is the total number of genes (background). Since many annotation sets were tested, we routinely corrected for multiple hypothesis testing (*Q*-values) using the False Discovery Rate procedure within each type of annotation set (14). For functional testing, we used the ConsensusPathDB tool (version 32) which holds 5068 pre-defined pathway gene sets along with the latest GO terms (15). Pre-defined gene lists from literature (16) included 4227 genes that were found monoallelically, and 6006 genes that were found biallelically expressed across multiple cell lines, 226 genes reported to evolve under balancing selection (BS), 104 genes with ancient derived protein-coding polymorphisms or haplotypes predating the human-Neanderthal split (HNS) and 60 genes with any evidence of human-chimpanzee trans-species polymorphisms or haplotypes (TSPs).

### Protein structure and missense variant mapping

Genomic positions of PFA-nsSNPs were submitted to the MuPIT web server (17) and mapped to proteins for which crystal structures (PDB references) are available. Tertiary structures were downloaded from the PDB database (18) and physical distances between amino acid changes were computed with the Chimera software (19).

## RESULTS

### Global abundance of *cis* configurations of coding variants

*Cis* configurations of functionally significant variants, leaving one form of the gene intact, would be expected to occur more frequently in human genomes to preserve organismal function (6). To test this hypothesis in 1092 genomes (1), we determined the ratio of *cis* to *trans* configurations of PFA-nsSNPs across all autosomal protein-coding genes for each of the genomes and derived the median of these ratios (Methods). In fact, highly significant abundance of *cis* configurations of PFA-nsSNPs was obtained, with a global *cis/trans* ratio of 59.6:40.4 (*P* < 3.53 × 10^−21^) (Table 1). Significant *cis* abundance was moreover apparent in each of the four ancestry groups contained in the 1092 genomes (1), with the *cis* fractions being similarly high in EUR (61.2%; *P* < 2.25 × 10^−21^), EAS (59.5%; *P* < 1.46 × 10^−17^) and AMR (60.1%; *P* < 3.09 × 10^−20^) and lower in AFR (54.7%; *P* < 1.66 × 10^−14^). An excess of *cis* configurations was also evident when examining the entirety of nsSNPs, with nearly identical results obtained from the total of 1092 genomes as well as each of the four ancestry groups alone (Table 1). Similar *cis*-excess was observed for all sSNPs contained in the coding sequences (Table 1). *Cis* abundance of PFA-nsSNPs was also evident when analyzing each of the 14 populations separately that were contained in the 1092 genomes, with their *cis/trans* ratios being nearly identical with the median of their respective ancestry groups, with the exception of population ASW in AFR, its *cis* fraction of 56.3% slightly exceeding the AFR median (Supplementary Table S1). When analyses were expanded to the entirety of nsSNPs and sSNPs, the results were once more highly congruent (Supplementary Table S1). Importantly, significant *cis* abundance was cross-validated by the analysis of the 184 experimentally phased PGP genomes, with nearly identical ratios of 60.4:39.6 (*P* < 1.66 × 10^−16^) for PFA-nsSNPs (Methods), and ratios of 63:37 (*P* < 4.37 × 10^−51^) and 65.1:34.9 (*P* < 7.80 × 10^−70^), respectively, for the totals of nsSNPs and sSNPs (Table 1). In sum, these results show that protein function-altering nsSNPs, and coding variants as a whole, are in effect not distributed randomly between the two homologues of a gene, but occur significantly more frequently on one of the two gene forms. On average, approximately 60% of the mutated autosomal genes in each genome have their coding variants in *cis*, and approximately 40% in *trans*.

**Table 1.** Global abundance of *cis* configurations of coding variants

Population samples^a^	No. phased genomes^a^	*Cis* configs PFA-nsSNPs^b^ (%)^c^	*Trans* configs PFA-nsSNPs^b^ (%)^d^	*P*-value^e^	*Cis* configs nsSNPs^f^ (%)^c^	*Trans* configs nsSNPs^f^ (%)^d^	*P*-value^e^	*Cis* configs sSNPs^g^ (%)^c^	*Trans* configs sSNPs^g^ (%)^d^	*P*-value^e^
1000G	1092	59.6	40.4	3.53 × 10^−21^	59.7	40.3	6.16 × 10^−51^	62.3	37.7	1.89 × 10^−71^
EUR	379	61.2	38.8	2.25 × 10^−21^	60.7	39.3	4.84 × 10^−52^	62.9	37.1	1.55 × 10^−71^
EAS	286	59.5	40.5	1.46 × 10^−17^	60.2	39.8	2.99 × 10^−45^	62.9	37.1	4.56 × 10^−65^
AMR	181	60.1	39.9	3.09 × 10^−20^	59.5	40.5	3.34 × 10^−50^	62.4	37.6	7.15 × 10^−71^
AFR	246	54.7	45.3	1.66 × 10^−14^	53.5	46.5	2.43 × 10^−30^	55.0	45.0	4.60 × 10^−43^
PGP	184	60.4	39.6	1.66 × 10^−16^	63.0	37.0	4.37 × 10^−51^	65.0	35.0	7.80 × 10^−70^

^a^1092 statistically haplotype-resolved genomes from the 1000 Genomes (1000G) Consortium including the four different ancestry groups EUR, EAS, AMR, AFR described by Abecasis *et al.* (1); 184 experimentally haplotype-resolved genomes from the Personal Genome Project (PGP) described by Mao *et al.* (7).

^b^Predicted protein function-altering non-synonymous SNPs (PFA-nsSNPs) from 1000G annotation database (1), or annotated using a combination of PolyPhen-2 (9), SIFT (10) and GERP conservation scores (11) in the PGP genomes.

^c^Values represent the median of *cis* fractions (%), i.e. the number of *cis* configurations divided by total configuration count per genome across the genomes.

^d^Analogous to^c^, *trans* fractions (%) being 100%–*cis* fractions (%).

^e^Exact Binomial test (see Materials and Methods; Hoehe *et al.* (6); *cis* counts were tested against the null hypothesis with a (composite) probability *P* = 0.4 for a *cis* configuration.

^f^nsSNPs from 1000G annotation database (1) or the PGP database, respectively.

^g^Synonymous SNPs (sSNPs) from 1000G or PGP database.

Configs, configurations.

### More *cis* than *trans* configurations in virtually all individual genomes

**Figure 1.**
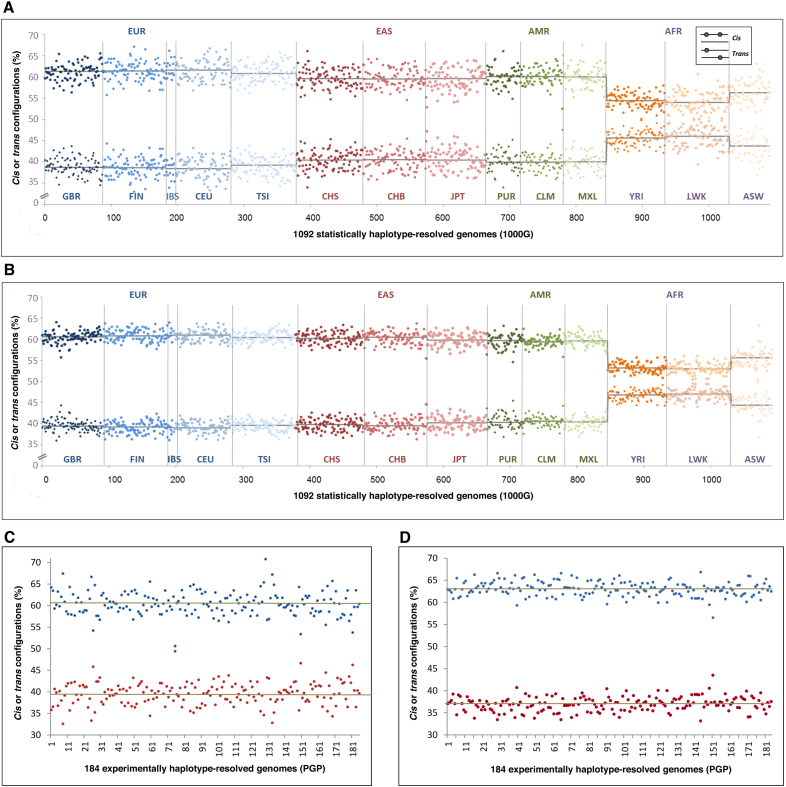
Individual fractions of *cis* and *trans* configurations of coding variants in 1000 Genomes and PGP. (**A**) Results shown for predicted protein function-altering nsSNPs (PFA-nsSNPs). The fractions of *cis* configurations (%) (number of *cis* configurations divided by total configuration count per genome) (*y*-axis) are presented in the upper half, the complementary *trans* fractions (100%–*cis* fraction (%)) in the lower half. Results are shown for each of the 1092 statistically haplotype-resolved genomes from the 1000 Genomes (1000G) database (1) (*x*-axis), ordered by ancestry group (as indicated on top; color-coded), and further subdivided into different populations as indicated at the bottom (separated by vertical lines), color-coding according to Abecasis *et al.* (1); horizontal black lines indicate median values for each of these populations. (**B**) Correspondingly, individual fractions of *cis* and *trans* configurations from the analysis of all nsSNPs. (**C**) Individual fractions of *cis* (blue) and *trans* (red) configurations of PFA-nsSNPs shown for each of the 184 experimentally haplotype-resolved PGP genomes. (**D**) Correspondingly, PGP results from the analysis of all nsSNPs.

Inspecting the 1092 genomes individually, in fact, 99.7% exhibited more *cis* than *trans* configurations of PFA-nsSNPs. Individual *cis* fractions varied within a limited range, between 55.7 and 67.1% in EUR and at most between 52.5 and 67.4% in AMR (Figure 1A). Inspecting AFR, individual *cis* fractions were between 49.7 and 63.1%, with nearly half exceeding 55%. Testing each of the 1092 genomes for statistical significance of *cis* abundance using an exact binomial test (Methods), literally every single genome exhibited significantly higher *cis* fractions than would be expected if the variants were distributed randomly between the two homologues of a gene (*P* < 2.55 × 10^−29^–2.30 × 10^−10^ in EUR, EAS and AMR and 8.83 × 10^−23^–1.32 × 10^−07^ in AFR). Examining the *cis* and *trans* configurations that were constituted by the entirety of nsSNPs, the results were very similar (Figure 1B): *cis* abundance again was highly significant in each of the 1092 genomes (*P* < 1.04 × 10^−67^–2.38 × 10^−31^ in EUR, EAS and AMR; *P* < 4.76 × 10^−60^–3.68 × 10^−15^ in AFR). The same was true for individual genome *cis* fractions of coding sSNPs (*P* < 5.54 × 10^−94^–7.95 × 10^−53^ in AMR, EAS and EUR; *P* < 9.18 × 10^−69^–7.32 × 10^−27^ in AFR). The seemingly lower variance of *cis* and *trans* fractions between individual genomes in the case of nsSNPs and sSNPs was apparently due to the roughly 3-fold higher number of phase-sensitive genes per genome in these cases. Also each of the 184 experimentally haplotype-resolved PGP genomes exhibited significantly higher *cis* than *trans* fractions for each type of coding variants, with *P* < 2.54 × 10^−26^–7.99 × 10^−06^ for PFA-nsSNPs, with *cis* fractions between 49.4 and 70.8% (Figure 1C), *P* < 2.46 × 10^−61^–1.00 × 10^−38^ for the entirety of nsSNPs (Figure 1D), and *P* < 3.87 × 10^−93^–9.76 × 10^−42^ in the case of sSNPs. Notably, significant *cis* abundance was also obtained when analyzing these classes of coding variants together, i.e. PFA-nsSNPs, nsSNPs and sSNPs as they co-occur in many genes (*cis/trans* ratio 58.2:41.8, *P* < 3.38 × 10^−89^). In sum, these results strongly support the assumption that significant abundance of *cis* configurations of coding variants represents a universal characteristic of diploid human genomes. Supported by the decay of *cis* fractions in AFR, *cis* abundance of coding variants most likely reflects a yet unrecognized manifestation of LD, ‘another face of LD’, as a common, fundamental feature of genome variation.

### *Cis* abundance primarily driven by pairs of coding variants

The reported *cis/trans* ratios were obtained from genes with different numbers of variants. Because the probability of variants to occur in a *trans* configuration increases with the number of variants in a gene, these ratios essentially represent composite values. Therefore we dissected these further, assessing the *cis/trans* ratios separately for configurations with 2 up to 5 variants, which accounted for 95.7% of all configurations in the case of PFA-nsSNPs, and similar fractions in the case of nsSNPs and sSNPs, respectively. By far the most frequent configurations of PFA-nsSNPs, 66.7%, were found to consist of pairs of these variants, 65.8% of which resided in *cis*. The second most frequent configurations, 18.6%, were combinations of 3 PFA-nsSNPs, occurring in equal proportions in *cis* or *trans*. As expected, the fractions of *trans* configurations increased to 53.4% in the case of 4 and 60.3% in the case of five PFA-nsSNPs, accounting, however, for only small fractions of all configurations, 7.3 and 3.1%, respectively (Supplementary Table S2A). Dissecting the composite *cis/trans* ratios separately in AFR, EUR, AMR and EAS showed an excess of pairs of PFA-nsSNPs also in each ancestry group (Supplementary Table S2A). Moreover, pairs of PFA-nsSNPs accounted for roughly two third of all configurations in the PGP genomes, of which again two third were in *cis* (Supplementary Table S2A). Similar results were obtained when dissecting the *cis/trans* ratios calculated from the entirety of nsSNPs and sSNPs, and analyzing all types of coding variants together (Supplementary Table S2B). A more in-depth evaluation of significant *cis* excess, comparing observed and theoretically expected *cis/trans* ratios for defined numbers of variants as well as simulating phased genomes to derive expected composite ratios, is presented in Supplementary Results, Supplementary Methods, and Supplementary Table S3. In sum, the dissection of the composite *cis/trans* ratios shows that the abundance of *cis* configurations is primarily driven by genes with pairs of coding variants that predominantly reside in *cis*.

Further analyses of these pairs of PFA-nsSNPs in *cis* showed that they were much more closely spaced than pairs in *trans*, with inter-mutation genome distances of 1570 bp (median) as opposed to 5290 bp in EUR, 1830 versus 4771 bp in AFR and 2584 versus 5984 bp in PGP (Supplementary Table S4). The same pattern was observed for all pairs of nsSNPs and coding sSNPs, respectively, though absolute distances were much larger (more detailed information in Supplementary Results andSupplementary Table S4). Moreover, inter-mutation genome distance was directly inversely related with *cis/trans* ratio; the smaller the distance, the higher the fraction of *cis* configurations. Thus, for instance, the smallest average inter-mutation distance in EUR, 11 bp, corresponded to the highest *cis/trans* ratio, 77:23, which declined to 51:49 at a distance of 54 281 bp (Supplementary Figure S2A) (more detailed information in Supplementary Results andSupplementary Figure S2A–H). In sum, significant *cis* abundance is primarily due to an excess of pairs of coding variants in *cis* that are closely spaced, while pairs of coding variants farther apart are more likely to reside in *trans*, suggesting that *cis* abundance is largely driven by distance.

Moreover, the pairs of PFA-nsSNPs in *cis* were more frequent than the pairs in *trans*. To this end, we determined the average minor allele frequencies (MAFs) per variant pair in both EUR and AFR. Notably, the PFA-nsSNPs occurring in pairs were found to exhibit very similar MAFs, which were provided by the 1000G database for each ancestry group, and combinations of common PFA-nsSNPs with singletons, for instance, were very rarely observed. To begin with, we calculated the mean of the average MAFs for all pairs of PFA-nsSNPs in *cis*, and *trans*; these were 0.18 and 0.16 in EUR, and 0.143 and 0.128 in AFR, respectively. Evaluating the average MAF spectra, the average MAFs per variant pair in either configuration peaked between 0.1 and 0.25, with a larger upper tail of *cis* compared to *trans* configurations (≥0.2: 64% in *cis* vs. 56% in *trans* in EUR; 46% in *cis* versus 35% in *trans* in AFR) (Supplementary Figure S3A and B). This is in striking contrast to the MAF spectra obtained from the entirety of (single) PFA-nsSNPs, where 56% of such variants in EUR (45% in AFR) had an MAF ≤0.01 (Supplementary Figure S3C). Taken together, these findings could reflect the result of ancestral admixture as a potential underlying mechanism. Accordingly, the observed, significant *cis* abundance results primarily from pairs of protein function-altering variants and coding variants as a whole that are closely spaced and therefore have been inherited together until present. Thus, they are more common than pairs of coding variants in *trans* which, much farther apart, have been subject to recombination, but also may be due to evolutionary forces other than recombination, such as genetic mutation and positive selection. So pairs of co-occurring protein function-altering variants in *cis*, which are even closer together than other pairs of coding variants in *cis*, may represent ancestral signals of potential functional significance, and mark small ancestry segments in the ‘mosaic that is the human genome’ (20).

### A global set of phase-sensitive genes

To explore the potential functional significance of *cis* abundance, we moved from whole genome analysis to the genes underlying this phenomenon. Specifically, we focused on the genes with coding variants predicted to affect protein function, where phase is most likely to have an impact. To begin with, the numbers of phase-sensitive genes and their *cis* and *trans* forms were highly similar between the genomes (for comprehensive numerical characterization and numerical relationships to PFA-nsSNPs and nsSNPs in a genome see Supplementary Results; Supplementary Table S5A and B; Supplementary Table S6A–D). We thus asked further: To what extent are the genes in each of these categories the same? Is there a common, shared set of phase-sensitive genes? Can we further distinguish within such a set two groups of genes, which preferentially have either *cis*, or *trans* configurations?

**Figure 2.**
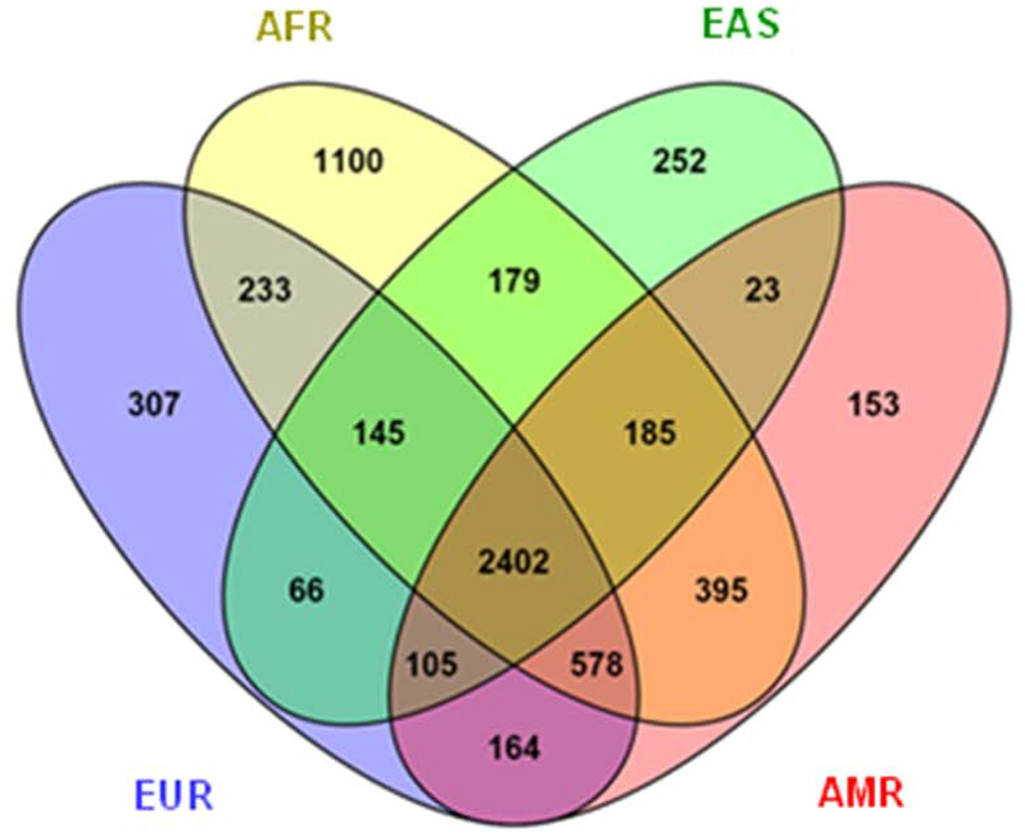
A global set of phase-sensitive genes. Venn diagram including the set of 2402 phase-sensitive genes (with ≥2 predicted protein function-altering nsSNPs (PFA-nsSNPs)) as shared by all four ancestry groups AFR, EAS, AMR and EUR (color-coded) from the 1000 Genomes database. In addition, the numbers of phase-sensitive genes that are common to any three or two of these ancestry groups are indicated as well as those that are private to one of these groups.

In fact, we were able to identify a set of 2402 genes with ≥2 PFA-nsSNPs, which were shared by all ancestry groups (Figure 2; Supplementary Table S7). To this end, we intersected the genes which were phase-sensitive in each of the ancestry groups, 4000 in EUR, 3357 in EAS, 4005 in AMR and 5217 in AFR, any two of which overlapped by 80–87%. This ‘global set of phase-sensitive genes’ unveiled a highly significant overrepresentation of manifold GO terms (Methods) including receptor activity, mainly olfactory, trans-membrane signaling and G-protein coupled receptor (GPCR) activity, the detection of (chemical) stimuli and sensory perception, membrane- and extracellular matrix (ECM)-related components/molecules, cell adhesion, the binding of odorants and antigens, multiple activities involved in the metabolism of substrates and xenobiotics, and MHC proteins (*P* < 6.12 × 10^−46^–9.56 × 10^−04^ for the top 99 GO groups) (Supplementary Table S8A). These results were complemented by a highly significant enrichment of pathways (Methods) including the transduction of olfactory signals, GPCR signaling and other types of signal transduction, ECM organization/receptor interactions, cell surface interactions, numerous immune-related processes such as antigen processing, Graft-versus-host and autoimmune diseases as well as multiple other diseases, chemical carcinogenesis, numerous biosynthetic and metabolic pathways, e.g. xenobiotics, drug metabolism and transport (*P* < 1.31 × 10^−52^–9.4 × 10^−03^ for the top 61 pathways; Supplementary Table S8B). 68% of the genes (1627) contained in the global set were also found to be phase-sensitive in the 184 experimentally phased PGP genomes. These were significantly enriched for similar biological functions: PGP genes shared 78% of the overrepresented GO terms (*P* < 1.30 × 10^−41^–0.001) and 76% of the pathways (*P* < 6.03 × 10^−46^–0.01) with the global set (Supplementary Table S8C and D; Supplementary Figure S4A and B), allowing extraction of essentially the same biological results. Taken together, there exists a common, global set of phase-sensitive genes encoding two different, potentially functionally distinct homologues. These may modulate cell-cell communication, cell-environment interactions and membrane-related processes, immune processes, metabolism and biosynthesis, and the development of disease.

This global set is part of a larger global set of 7524 genes with ≥1 PFA-nsSNPs (‘variable genes’) (Supplementary Table S7). These were similarly enriched for these functional categories (Supplementary Table S9A and B); the genes with ≥2 PFA-nsSNPs, however, made a disproportionately larger contribution to the pathways and GO terms in question (Supplementary Figure S5A and B, more detail in Supplementary Results). In contrast, 5040 genes (Supplementary Table S7) did not contain any PFA-nsSNPs at all in the 1092 genomes and were significantly enriched for numerous essential cellular functions which may not tolerate variability (*P* < 6.15 × 10^−25^–9.51 × 10^−11^ for the top 50 pathways; *P* < 2.35 × 10^−24^–2.12 × 10^−14^ for the top 50 GOs) (Supplementary Table S10A and B). Thus, the PFA-nsSNPs giving rise to the global sets of phase-sensitive and variable genes could result from processes of selection to maintain the long term diversity of these genes and preserve their functional flexibility to allow adaptation of the organism to internal and external stimuli. In fact, predominantly the global set of phase-sensitive genes, but also the global set of genes with ≥1 PFA-nsSNPs were significantly enriched with genes reported to be under balancing selection (BS) (*P* = 9.29 × 10^−07^ and *P* = 4.08 × 10^−03^, respectively) (16). Moreover, both global sets were significantly enriched for monoallelically expressed (MAE) compared to biallelically expressed (BAE) genes (Methods; *P* = 1.75 × 10^−11^ and 1.99 × 10^−14^, respectively) (16), further corroborating their potential role as major modulators and adaptive agents. Similarly to MAE, they were significantly enriched with genes with ancient derived protein-coding polymorphisms or haplotypes predating the human-Neanderthal split (HNS) (Methods; *P* = 3.09 × 10^−16^ and *P* = 4.77 × 10^−12^, respectively) (16). Finally, the over-representation of evolutionarily significant gene sets was even stronger in the 1627 phase-sensitive genes cross-validated by PGP, with *P* = 3.79 × 10^−22^ for HNS, *P* = 1.23 × 10^−09^ for BS, and *P* = 6.56 × 10^−2^ for genes with evidence of human-chimpanzee trans-species polymorphisms (TSP) (Supplementary Figure S6). Having demonstrated existence of a shared, global set of phase-sensitive genes underlying *cis* abundance, can we further distinguish within this set two groups of genes, which have preferentially either *cis*, or *trans* configurations of protein function-altering coding variants?

### *Cis*- and *trans*-abundant genes

To this end, we evaluated the gene-based *cis* and *trans* fractions for each of the 2402 phase-sensitive genes across the entirety of 1092 genomes using a Binomial test (Methods). In fact, we identified a subset of 1227 genes exhibiting significantly more frequently *cis* configurations (*P* < 1.05 × 10^−63^–0.048), which we defined as ‘*cis*-abundant genes’, and 786 genes having significantly more frequently *trans* configurations (*P* < 1.78 × 10^−15^–0.049) defined as ‘*trans*-abundant genes’ (Supplementary Table S11A and B). These genes were present in each of the ancestry groups, with the same ‘configuration type’. Thus, *cis* or *trans* abundance could represent a constant characteristic of a gene. Taken together, the vast majority (84%) of the autosomal genes with ≥2 PFA-nsSNPs that were contained in the global set can be classified into the two major categories *cis*- and *trans*-abundant genes, while the remainder, 385 genes, had nearly equal proportions of both configurations. This classification was further validated including the population-specific phase-sensitive genes per ancestry group (Figure 2) in analysis, and assessing the PGP genomes (more detail in Supplementary Results; for distribution of *cis*- and *trans*-abundant genes across the autosomes see also Supplementary Figure S7A and B, and for corresponding autosomal *cis/trans* ratios see Supplementary Table S12). In sum, 78–88% of all phase-sensitive genes were classifiable in each of the sample sets examined. Importantly, the group of *cis*-abundant genes was always larger than that of *trans*-abundant genes, with ratios of 1.55:1 to ∼2:1, resulting in significant global *cis* abundance as the net effect.

These two gene categories were distinctively enriched for over 75% of the pathways which were found overrepresented in the global set: *cis*-abundant genes for signaling by GPCR and signal transduction, biosynthetic and especially metabolic pathways, e.g. xenobiotics metabolism including cytochrome P450-mediated oxidation and other phase 1-related processes (*P* < 1.81 × 10^−51^–0.008); *trans*-abundant genes for numerous immune response-related processes and diseases as well as autoimmune diseases, viral and infectious diseases, cell surface interactions, ECM-receptor interaction, signaling pathways, and drug transport (*P* < 3.26 × 10^−7^–0.009) (Supplementary Table S13A and B; Supplementary Figure S8A). Thus, the disease-related phenotypes found enriched in the global set appear to be primarily mediated by *trans*-configurations. These two gene categories were furthermore differentially enriched for 57–83% of the GO terms, evaluated separately for their taxonomies: *cis*-abundant genes for instance for GPCR activity and signaling, odorant binding, and activities involved in the metabolism of substrates; *trans*-abundant genes for instance for MHC proteins/receptors, antigen binding, ECM structural constituent and organization, and cell adhesion (Supplementary Table S13C and D; Supplementary Figure S8B). In sum, *cis* and *trans*-abundant genes may be differentially involved in gene functions and pathways, potentially reflecting different mechanisms to exert gene functions.

**Figure 3.**
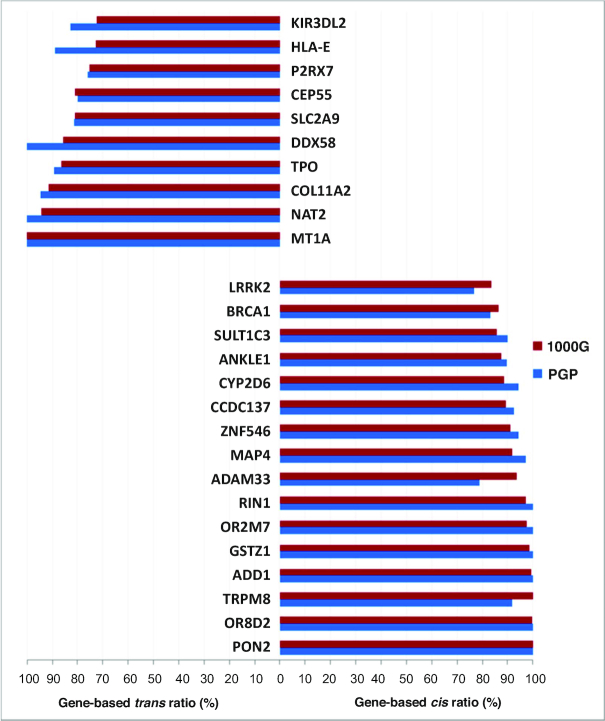
Examples of *cis*- and *trans*-abundant genes. Genes were selected from the cross-validated sets of 322 *cis*- and 153 *trans*-abundant genes, which were shared by both 1000 Genomes (1000G) and PGP. Top left *trans*-abundant genes, lower right *cis*-abundant genes; blue bars indicate the gene-based *cis*- and *trans* fractions (%) derived from the 184 experimentally phased PGP genomes, red bars the corresponding fractions derived from the 1092 statistically phased genomes (1000G).

Subsequently we tested the validity of concrete *cis*- and *trans*-abundant genes identified in the global set against the experimentally phased genomes. Intersecting the 1227 *cis*-abundant genes that were shared by all ancestry groups in 1000G, with the 778 genes *cis*-abundant in PGP resulted in an overlap of 322 genes. Correspondingly, 153 of the 786 *trans*-abundant genes were also observed in PGP, a sample only one-sixth part of the size. Figure 3 shows examples of these cross-validated genes, demonstrating that statistically and experimentally phased genomes essentially deliver the same results and, moreover, illustrating the key point that the majority of autosomal genes with ≥2 PFA-nsSNPs have either *cis* or *trans* configurations in significant excess. Thus, notable fractions of *cis*- and *trans*-abundant genes exhibited solely *cis* or *trans* configurations, respectively, i.e. gene-based *cis* or *trans* fractions of 100%, such as *PON2, OR8D2* or *MT1A* (Figure 3) (for detailed quantitative characterization of gene-based *cis* and *trans* fractions see Supplementary Results). A more expanded analysis validated that *cis* and *trans* abundance in effect represents a constant characteristic in nearly 90% of the genes with only 8.7% of all *cis*- and 12.9% of all *trans*-abundant genes changing configuration type in PGP (more detailed information in Supplementary Results).

Examples of *cis*-abundant genes (Figure 3) include members of pathways and GOs that were significantly overrepresented in this gene category, such as *CYP2D6, GSTZ1, MAP4* and *SULT1C3* involved in xenobiotics metabolism, or *OR8D2* and *OR2M7* involved in olfactory transduction. Numerous *cis*-abundant genes have conserved protein domains, such as *ZNF546*, a C2H2 zinc finger gene. Moreover, some *cis*-abundant genes represent members of gene families, which as a whole are ‘*cis*-abundant’, i.e. their members are significantly more often *cis*-abundant, such as the olfactory receptor (OR) gene family (*P* = 3.41 × 10^−05^) (which overall contributes 0.87% to the global *cis* fraction of ∼60%). Examples include moreover disease genes such as *BRCA1, LRRK2, ADAM33, ADD1* or *PON2*. Examples for *trans*-abundant genes (Figure 3) include genes involved in immune response, immune and autoimmune diseases and viral and infectious diseases, such as *HLA-E, KIR3DL2, TPO* and *DDX58*, genes involved in cancer such as *MTA1* and *NAT2*, and genes involved in pathways and GOs enriched in this category, such as *P2RX7*. Some of these *trans*-abundant genes, too, are members of predominantly *trans*-abundant gene families, such as the histocompatibility complex (*P* = 2.49 × 10^−03^), and collagen family (*P* = 1.26 × 10^−02^).

**Figure 4.**
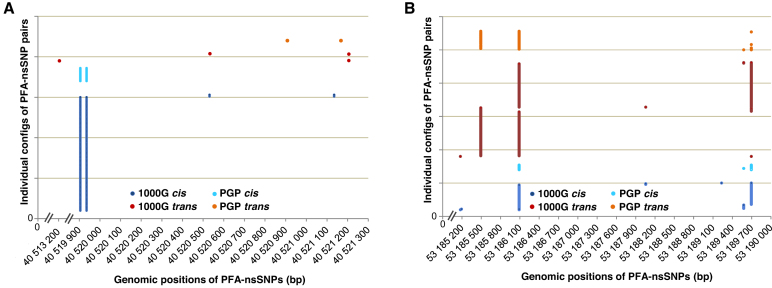
Typical distributional patterns of pairs of predicted protein function-altering nsSNPs (PFA-nsSNPs) in *cis*- and *trans*-abundant genes. The specific configurations of pairs of PFA-nsSNPs, specified by genome positions (bp), are presented as observed in 1000G and PGP. Each individual pair of PFA-nsSNPs is indicated by a pair of solid circles, color-coded by source. (**A**) Distributional pattern of PFA-nsSNPs in the *cis*-abundant *ZNF546* gene. The same pair of PFA-nsSNPs in *cis* occurs in numerous individual genomes in both 1000G (blue) and PGP (light blue) and therefore appears as two vertical, parallel lines; another pair of PFA-nsSNPs in *cis* is observed in 1000G twice, and two pairs of PFA-nsSNPs in *trans* which share the rightmost variant; one pair of PFA-nsSNPs in *trans* is observed in PGP. (**B**) Distributional pattern of PFA-nsSNPs in the *trans*-abundant *KRT3* gene. Apparently, the pairs of PFA-nsSNPs are less frequent; one pair of PFA-nsSNPs occurs in *trans* in both 1000G and PGP (left); another pair of PFA-nsSNPs (its left variant shared with the other pair) occurs in *trans* in 1000G; the same pair also shows a less frequent, though still sizable fraction of *cis* configurations in both 1000G and PGP, consistent with a change from *cis* to *trans* for more distant pairs of PFA-nsSNPs.

**Figure 5.**
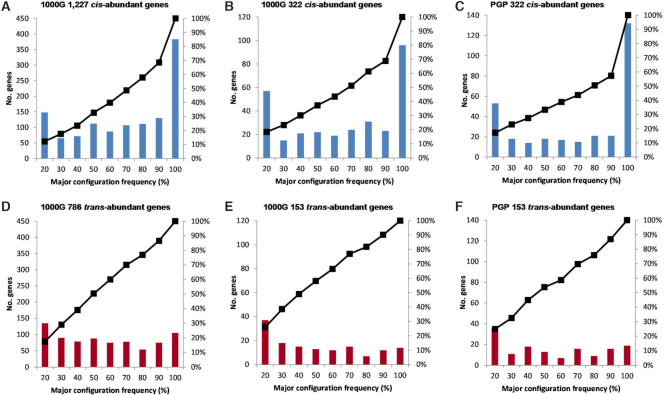
Major configuration frequencies in *cis*- and *trans*-abundant genes. The ‘major configuration’ of a *cis*- or *trans*-abundant gene is defined as the pair of predicted protein function-altering nsSNPs (PFA-nsSNPs) that exhibits the highest number of *cis, or trans* counts, respectively, relative to the total of *cis or trans* counts observed for this gene in a population sample. This ratio is defined as the ‘major configuration frequency’ (MCF) of a gene. The MCFs of defined numbers of genes are binned in 10% intervals. They were assessed for 1227 *cis*- and 786 *trans*-abundant genes in 1092 genomes from the 1000 Genomes (1000G) database, and for the cross-validated sets of 322 *cis*- and 153 *trans*-abundant genes shared by both 1000G and PGP. (**A**) Blue bars indicate the numbers of *cis*-abundant genes (*y*-axis left) which have a major configuration with a frequency (%) as specified by given bins (*x*-axis). For example, referring to the highest blue bar on the right, 383 *cis*-abundant genes have a major configuration with a frequency between >90 and 100% of total *cis* count. The black graph represents the cumulative percentage (%) of the number of these genes (*y*-axis right). (**B**) Accordingly, MCFs binned for 322 *cis*-abundant genes from 1000G and (**C**) for the identical 322 *cis*-abundant genes assessed in the 184 experimentally phased PGP genomes. The corresponding results for *trans*-abundant genes (red bars) are presented in (**D**), (**E**) and (**F**).

Subsequently we evaluated the specific genomic positions of pairs of PFA-nsSNPs in *cis-* versus *trans* to test for potentially functionally relevant, different distributional patterns. Inspecting several cross-validated genes, we observed striking differences. Thus, in *cis*-abundant genes such as *ZNF546* (Figure 4A), or *ADAM33, PON2, ZNF626* and *KRT83*, one pair of (closely spaced) PFA-nsSNPs dominated the picture. Remarkably, the two coding variants establishing such a ‘major configuration’ nearly always occurred together, and only rarely, one of the two PFA-nsSNPs occurred alone, or in combinations with other coding variants. In some instances, few other pairs or combinations of PFA-nsSNPs in *cis* or *trans* were observed (Figure 4A). A much more mixed picture was observed in *trans*-abundant genes, as exemplified by *KRT3* (Figure 4B). This was characterized by two or more, apparently less frequent pairs of PFA-nsSNPs in *trans*; in this example, a *trans* pair existed to a smaller extent still in *cis*. To test whether these case observations represent a more general picture, we examined the 1227 *cis*- and 786 *trans*-abundant genes identified in the global set, and the 322 *cis-* and 153 *trans*-abundant genes shared by both 1000G and PGP. We determined the proportion of the most frequent pair of PFA-nsSNPs relative to its corresponding total of configurations for each of the genes, across the genomes. Then we binned the genes by major configuration frequency (%). As shown in Figure 5A, a substantial fraction, 383 of the 1227 *cis*-abundant genes (31.2%) exhibited one major pair of PFA-nsSNPs accounting for > 90 up to 100% of all *cis* configurations, and 42% one major pair accounting for >80%. This result was strongly confirmed in the cross-validated genes (Figure 5B and C), and is in agreement with the average MAF spectra of pairs of PFA-nsSNPs in *cis* described earlier. A strikingly different picture was observed for *trans*-abundant genes, characterized by the absence of a major configuration which would be present in sizable fractions (Figure 5D–F). For example, the frequency of the major configuration with MCFs >90% is more than twice as high for *cis*-abundant genes (e.g. 383 out of a total of 1227 in 1000G) compared to the corresponding *trans*-abundant genes (101 out of 768) (Figure 5A and B, left), with relative frequencies of 0.31 versus 0.13. The differences are even more pronounced in the cross-validated gene sets. In sum, significant *cis* abundance is based on a considerable fraction of *cis*-abundant genes, which have highly frequent pairs of protein function-altering variants co-occurring on the same homologue. Thus, we have traced *cis* abundance back to specific, relatively ancient pairs of potentially functionally significant coding variants in *cis*.

**Figure 6.**
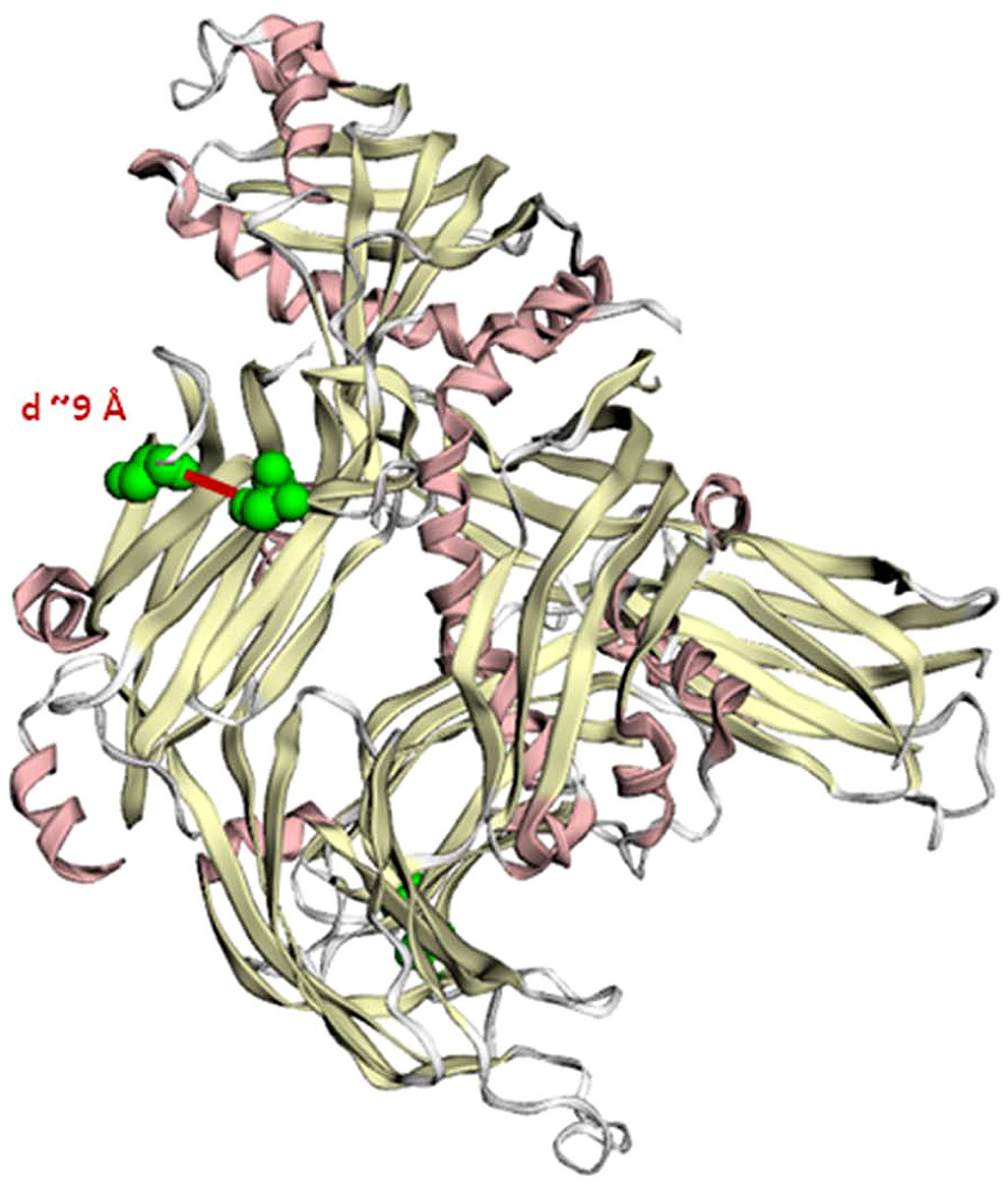
Major configuration in a *cis*-abundant gene mapped onto protein tertiary structure. Two neighboring amino acid changes predicted to affect protein function in *cis* (S584P and S610G) in the protein ESYT2 (Extended Synaptotagmin 2) are shown. Both amino acid substitutions fall in the C2 domain that targets the protein to the cell membrane. They are located within a physical distance of ∼9 Å. Tertiary structure was taken from the PDB database (ID: 4P42) (18). Mapping was generated with the MuPIT web server (17). Residue distance was computed with the Chimera software (19).

Attempting to interpret this finding, we considered epistatic interactions between these pairs of coding variants, a potential role as compensatory deviations, or co-evolution. In order to explore whether these major, closely spaced pairs of variants could in fact indicate potential interactions between the corresponding amino acid substitutions, we have mapped them onto protein tertiary structures using the Protein Data Bank (PDB) (18). Indeed, for some proteins, for example ESYT2 (Extended Synaptotagmin 2), a membrane-related protein, we found that the corresponding residue pairs existed in close physical distance (∼9 Å) (Figure 6). This could suggest possibility of physical interaction and/or functional interdependence, for instance indicating coevolution (21). To round up, we have obtained first, very preliminary evidence for potential functional implications of major pairs of coding variants in *cis*, the hallmark of *cis* abundance.

## DISCUSSION

This work represents the first large scale analysis of both statistically and experimentally haplotype-resolved genomes, 1276 in total from different populations, allowing establishment of common patterns of phase as key characteristics of diploid human genomes. Virtually all genomes showed significant abundance of *cis* configurations of functionally significant coding variants, at an overall global *cis/trans* ratio of ∼60:40. The vast majority of genes exhibited either *cis*, or *trans* configurations in significant excess, allowing distinction of *cis*- and *trans*-abundant genes as major categories of autosomal protein-coding genes. These patterns were largely constituted by a shared, global set of phase-sensitive genes that exist in either *cis* or *trans* configurations. The potential functional importance of these patterns was suggested by significant enrichment of this global set with gene sets indicating its involvement in adaptation and evolution; further by distinction of the functional classes concerned into those that are primarily mediated by *cis*-, or *trans*-abundant genes.

It is unlikely that these patterns represent an artifact due to bias in phasing. This statement is supported by (i) the high quality of 1000G statistical haplotype data and their strong validation by analysis of a substantial number of experimentally phased genomes; and (ii) the robustness of the experimental phasing method, ensured by an exceedingly low phasing switch error rate (9). Thus, additional steps to eliminate potential artifacts as causes of the *cis* and *trans* patterns of heterozygous variants, such as independent calculation of LD values from un-phased data, do not appear warranted. While some variation in the absolute numbers of *cis* and *trans* configurations of PFA-nsSNPs has been observed, possibly due to differences in population structures and size, or annotation and validation procedures, the relative fractions of *cis* and *trans* configurations proved to be quite stable. Moreover, it appears unlikely that the *cis/trans* ratios of PFA-nsSNPs were biased by the annotation algorithms used, as entirely different wet lab and bioinformatics pipelines, as well as control analyses of all nsSNPs and sSNPs produced highly similar ratios. Thus, the overall internal consistency of our data suggests that the key results can be considered robust and not an artifact of the specific algorithms and settings employed, or the parameters used.

This work represents an advance in the field of haplotype analysis in that an experimental phasing method (9) has been applied at large scale to address ‘higher order questions’ concerning the diploid nature of the human genome (3,4,22,23). Recent efforts have mainly focused on refining and improving technologies for the efficient haplotyping, generally demonstrated on small numbers of genomes. Major initiatives focusing on haplotype analysis, such as the HapMap and 1000 Genomes Projects have primarily been motivated to infer the position of disease variants through LD (1,24). In contrast, this work has addressed the potential functional implications of phase information to more fully understand diploid gene and genome function (3,4). In effect, *cis* abundance of protein function-altering variants and coding variants as a whole, represent another, yet unrecognized, manifestation of LD in the human genome, and the relationship between the extended haplotype blocks in the HapMap and the phased exonic sequences remains to be determined. Essentially, our work shows how existing patterns of LD in the human genome (25,26) affect the distribution of genetic coding variants between the two homologous chromosomes of autosomal genes. In this context, it is important to note that due to existing LD, a ∼50:50 *cis/trans* ratio of sSNPs and nsSNPs (i.e. coding variants which are not predicted to affect protein function) cannot be expected to underscore the functional significance of *cis* abundance of PFA-nsSNPs. This issue is related to the challenge of distinguishing the causative variants from those in LD in the context of disease gene identification. Importantly, analogous analyses which we performed solely with nsSNPs other than those predicted to alter protein function, and sSNPs, failed to generate the functional results obtained with the PFA-nsSNPs.

Moreover, this work represents a first comprehensive, phase-informed analysis of protein-coding genetic variation in diploid human exomes. Recent exome studies have mainly focused on characterizing the functional spectrum of allelic variants to facilitate the identification of disease-causing variants (2,27–31), and issues of phase have only barely been addressed. Our data have shown that, of a total of 18 121 autosomal genes (RefSeq), nearly one third (6284) have ≥2 PFA-nsSNPs in a fraction of the 1092 genomes and could be either *cis*- or *trans*-abundant. Moreover, nearly half of all coding variants predicted to alter protein function in a genome (and over 60% of all nsSNPs) were found to exist in either *cis* or *trans* configurations (Supplementary Table S6B and D). Phase information is moreover indispensable in human exomes because there are many genes with mono-allelic (32) or allele-specific expression, especially those modulated by epigenetic phenomena (33). In this case, the unique distribution of coding variants across the two chromosomal homologues affects which variants will ultimately have functional consequences. Thus, phase is an important link to differentially expressed transcriptomes and proteomes.

In fact, the global set of phase-sensitive genes and with it, *cis*- and *trans*-abundant genes, potentially encoding two functionally distinct homologues, have shown strong overlaps with MAE. This also refers to the specific (functional) classes which were overrepresented as well as enrichment with evolutionarily significant gene sets and the shift of allele frequency distributions towards those consistent with common variation (i.e. greater allelic age on average) (16,34). Thus, these phase-sensitive genes, particularly in combination with MAE, may commonly confer functional flexibility, generating wide-spread cell-to-cell, organismal and phenotypic diversity. Their significant enrichment with functional classes involved in cell-cell and cell-environment interactions in all populations examined, moreover supports their general role in adaptation and evolution (6,16,22,35). In this context it is interesting to note that *cis*- and *trans*-abundant genes show a highly significant underrepresentation of genes with a high predicted probability of being haploinsufficient (36), with *P* < 1.0 × 10^−6^ for *cis*-abundant and *P* = 3.03 × 10^−5^ for *trans*-abundant genes (Supplementary Results; Supplementary Table S14A and B). This further corroborates the assumption that these genes possess adequate physiological function with one chromosomal homologue, underscoring potential haploid advantage (21). Future questions to be addressed are which of the two homologues of *cis*-, and *trans*-abundant genes are expressed, in spatial and temporal context. Future questions concern, overall, the role of diplotypic genes in transcriptome, proteome, and phenotype diversity within and between cells, tissues, individuals, populations, species, at various developmental and physiological stages, as well as health and disease. Moreover, future questions concern the impact of splicing on *cis*- and *trans* configurations. A first preliminary analysis of highly *cis*- and *trans*-abundant disease genes with known splicing variability, such as *BRCA1, TPO* and *TP53*, suggested that functionally significant coding variants within configurations could be linked to splicing events, in particular exon skipping (Supplementary Figure S9A–C). This could indicate a potential compensatory mechanism that will require further systematic investigation. Finally, an important question concerns the expressivity of disease variants in the context of *cis* and *trans* configurations. To take a first step in this direction we tested the entirety of protein function-altering nsSNPs that went into the *cis* and *trans* configurations contained in the global set against the OMIM and GWAS databases. In effect, 409 variants in OMIM were also present in functional variant combinations in a subset of 99 *cis*-abundant genes, and 329 OMIM variants were found in 67 *trans*-abundant genes. Similarly, small sets of GWAS SNPs were found in either gene category (Supplementary Table S15A–D). These results raise the question, to which extent the co-occurring variants can modify each other? Highlighting a potential importance of *cis*-genomic context, the analysis of compensatory interactions which could, for instance, cause a *cis*-suppression of pathogenic variants, is an interesting subject for future research.

This work has introduced two major categories of variable autosomal genes: *cis*- and *trans*-abundant genes. The more frequent *cis*-abundant genes are characterized by common pairs of closely spaced PFA-nsSNPs. Thus, these may represent ‘evolutionary signals’ which trace back to ancient populations. Reasons for their preservation could be epistatic or compensatory interactions between their corresponding amino acid substitutions, maintaining or enhancing the functionality of the protein (37–40), co-evolution (21), or hitchhiking effects (41). Evidence for potential physical interaction between such amino acid substitutions in *cis* configurations has been obtained in preliminary analyses at the example of ESYT2 (Figure 6). In this context, the observation of ‘coincident codons’ (42), invoking selection not only on protein, but also on the structure of the nucleic acid that encoded that protein, may further be of relevance. Once the (available) 3D structures in protein databases are more complete, these seemingly old, co-occurring pairs of protein function-altering coding variants discovered in this work, may provide valuable information for the study of protein evolution and functionality (38). *Trans*-abundant genes, on the other hand, apparently result from a mixture of mechanisms, such as the recombination of more distantly spaced coding variants (exemplified by pairs of variants, which exist to a small percentage still in *cis*, while to a larger extent in *trans*), the occurrence of mutations, or positive selection such as in the case of HLA genes (43). This latter example referring to relatively short genes illustrates that configuration status overall is not significantly influenced by gene length. While *trans*-abundant genes are on average longer than *cis*-abundant genes (primary transcript lengths 35 969 versus 25 859 bp, *P* = 3.9 × 10^−08^; protein lengths 1012 versus 875 amino acids; *P* = 0.001), corresponding with their much larger inter-mutation distances, the overall correlations between gene-based *trans* fractions and primary transcript (*c* = 0.07) as well as protein length (*c* = 0.01) evidently were not significant. Although there is an overall tendency for genes to reside in *trans* with increasing numbers of variants, the opposite was true for instance for the highly diverse OR genes, which were largely *cis*-abundant, their diversity being preserved by positive selection (43). Taken together, configuration status overall is neither the result of gene length nor the number of coding variants.

To further characterize *cis*- and *trans*-abundant genes, that is, diploid genes with either one or two variable homologues, novel approaches to analyze sequence-structure-function relationships will be required. These must allow the analysis of combinations of variants as compared to focusing on the likely functional effects of single nucleic or amino acid substitutions, both *in silico* and *in vitro*. As an advance over the presently available standard approaches, first sequence-based tools and inference methods have been reported which take as input a stretch of sequence that could have multiple variants in it, and are not necessarily restricted to a specific position (44–46). Likewise, *in vitro* approaches must go beyond the conventional single amino acid substitution testing assays, and routinely include the analysis of combinations of variants, i.e. molecular haplotypes. Moreover, novel approaches must account for three (different) states per gene, i.e. express and characterize the two molecular haplotypes of a gene separately, and jointly as a pair (4,6,23). The importance of proposed advances has been underscored by Drysdale *et al.* (47), who examined the molecular haplotypes of the human β_2_-adrenergic receptor gene and their pairs *in vitro*, and *in vivo* by assessing drug response. Their results indicated that ‘the unique interactions of multiple SNPs within a haplotype ultimately affect biologic and therapeutic phenotype’ and that ‘no isolated SNPs had any predictive power’. In sum, such forward-looking approaches should help to elucidate the differential influence of these two gene categories in a functional context. Further considerations concerning potential explanations for the phenomena described are included in the Supplementary Discussion.

This work represents a basis for diploid genomics (3), a ‘dual’ view of biological processes. It identifies, and focuses on, those sets of genes, the homologues of which are potentially functionally different (22), and divides those further into two categories, *cis*- and *trans*-abundant genes (i.e., diploid genes with either one, or two altered homologues). Thus, it sets the stage for a diploid biology which inherently is allelically biased at all levels: chromatin organization (48), epigenomes (33), transcriptomes including transcriptional regulation, and proteomes. Diploid biology will need to be described and understood with reference to the two homologues of the genes, regulatory sequences and proteins an individual possesses. In this context it should be pointed out what a challenge it is to presently describe the diploid nature of human genomes in words and terms that have been shaped by working with un-phased, i.e. ‘mixed diploid’ sequences leading to the perception and interpretation of a ‘one genome world’. Towards diploid genomics, major questions to be addressed in the future are, for example, whether, and how, biology could change with *cis/trans* ratios, or how phenotype could change with configuration status, and whether *cis* abundance could represent a general phenomenon in all diploid species.

## DATA AVAILABILITY

Read and mapping data for all PGP genomes reported here are available at the database of Genotypes and Phenotypes (dbGaP) under study accession number phs000905.v1.p1. In addition, the full data package minus reads and mappings are accessible through GigaDB as part of Mao *et al.*, 2016 (7).

## Supplementary Material

Supplementary Data
