# Supplementary material for: Significant abundance of cis configurations of coding variants in diploid human genomes
Source: Nucleic Acids Res. 2019 Jan 30;47(6):2981–95. doi: 10.1093/nar/gkz031 (PMC6451136; doi:10.1093/nar/gkz031)
Supplement: Supplementary Data [file gkz031_supplemental_files.zip › Hoehe_Supplementary_Data.pdf]

Significant abundance of *cis* configurations of coding variants in diploid human genomes

Margret R. Hoehe[1*], Ralf Herwig[1], Qing Mao[2], Brock A. Peters[2,3], Radoje Drmanac[2,3], George M. Church[4], Thomas Huebsch[1]

[1]Department of Computational Molecular Biology, Max Planck Institute for Molecular Genetics, 14195 Berlin, Germany

[2]Complete Genomics, Inc., San Jose, CA 95112, USA

[3]BGI-Shenzhen, Shenzhen 518083, China

[4]Department of Genetics, Harvard Medical School, Boston, MA 02115, USA

SUPPLEMENTARY MATERIAL

SUPPLEMENTARY RESULTS 2
SUPPLEMENTARY METHODS 5
SUPPLEMENTARY DISCUSSION 6
SUPPLEMENTARY FIGURES 6
SUPPLEMENTARY TABLES 17
SUPPLEMENTARY REFERENCES 27

SUPPLEMENTARY RESULTS

Observed versus theoretically expected *cis*/*trans* ratios, simulated expected composite ratios

To estimate the excess of *cis* configurations we compared observed and theoretically expected *cis*/*trans* ratios. The probability *P* for a configuration with a defined number of *n* variants to reside in *cis* is $1/2^{n-1}$, if the chance for every variant in a gene to occur on either homologue is equal. So for pairs of coding variants, the observed *cis*/*trans* ratios were between 66:34 and 70:30 versus expected 50:50; for combinations of 3 variants observed 50:50–54:46 versus expected 25:75, for combinations of 4 variants 45:55–47:53 versus 12.5:87.5, and for 5 variants 39:61–41:59 versus 6.25:93.75. This corresponded to a 1.32–1.4 up to 6.24–6.56-fold enrichment of *cis* fractions. In a second step, we generated 1,092 virtual, phased genomes, assigning to each variant in a gene a 50:50 chance to exist on either homologue (Supplementary Methods), and dissected the simulated data set accordingly. With the simulated *cis*/*trans* ratios being virtually identical for 2 up to 5 variants with the expected ones (as calculated above) (Supplementary Table S3), we were able to validly derive the expected composite *cis*/*trans* ratios, ~39:61 for predicted protein function-altering non-synonymous SNPs (PFA-nsSNPs), ~37:63 for nsSNPs and synonymous SNPs (sSNPs), respectively, and 33:67 combining all types of coding variants (Supplementary Table S3). Thus, *cis*/*trans* ratios observed ~60:40 versus expected below 40:60. So even where *cis* ratios are minimally below 50%, which is the case in three of 1,092 genomes, and one of 184 PGP genomes, they are still significantly higher than would be expected by chance.

Inter-mutation distances between pairs of coding variants in *cis* versus *trans* configurations

As evident from the detailed results in Supplementary Table S4, pairs of coding variants in *cis* extended, overall, over genome distances up to nearly 3,900 bp (median), and in *trans* up to nearly 8,000 bp in EUR and ~7,200 bp in AFR due to decay in LD. These intervals estimated in 1000G (1) were largely consistent with those in PGP (2), with the variants in *cis* spanning genome distances up to 3,562 bp, and the variants in *trans* spanning distances up to 8,280 bp. Notably, where pairs of PFA-nsSNPs in *cis* and pairs of sSNPs in *cis* were found to co-exist within a diploid gene, which was the case for roughly 12–15 genes per genome, they were found to reside on the same homologue in over 83% of cases, indicating localization within the same ancestral segment.

Relationship of inter-mutation genome distance with *cis*/*trans* ratio

Cis and *trans* configurations were sorted by inter-mutation genome distance (bp), binned per 6,000 configurations (i.e. distributed into approximately 20 bins), and an average inter-mutation distance with its corresponding *cis*/*trans* ratio assessed per bin. Accordingly, the smallest distance in EUR, 11 bp, corresponded to the highest *cis*/*trans* ratio, 77:23, which declined to 51:49 at a distance of 54,281 bp and fell marginally below 50% at the largest average inter-mutation distance calculated, 81,099 bp, where a cumulative *cis* fraction of 67.8% was reached (Supplementary Figure S2A and B). A similar inverse relationship between average inter-mutation distance and *cis*/*trans* ratio was observed in AFR, where an increase of distances from 14 to maximally 57,466 bp correlated with a (more rapid) decrease of *cis*/*trans* ratios from 75:25 to 48:52, with a cumulative *cis* fraction of 62.4% (Supplementary Figure S2C and D). These analyses, which refer to pairs of PFA-nsSNPs, were complemented by analogous analyses performed with the entirety of nsSNPs and sSNPs (Supplementary Figure S2E–H), strongly confirming this picture.

Numerical characterization of phase-sensitive genes and their *cis* and *trans* forms

How many genes have two or more PFA-nsSNPs, which could exist in either *cis* or *trans* configurations and constitute observed *cis* abundance? Each of the 1,092 genomes had between 393 and 710 such phase-sensitive genes (median 487), equivalent to 2.2–3.9% of all autosomal protein-coding genes (Hg19, RefSeq). In 221–397 (median 297) of these genes, the PFA-nsSNPs resided in *cis* and in 132–342 (median 193) in *trans* (Supplementary Table S5A); for corresponding results for each of the four ancestry groups see also Supplementary Table S5A, for corresponding numbers from all nsSNPs, which were overall ~2.7-fold higher and affected 5.7–10.3% of all autosomal genes, see Supplementary Table S5B. Taken together, the relatively low level of variation in the fractions of *cis* and *trans* configurations observed across the genomes corresponds to a relatively limited range of variation in the numbers of phase-sensitive genes, and their *cis* and *trans* forms. These numbers were

directly proportional to the numbers of PFA-nsSNPs and the entirety of nsSNPs in a genome across all ancestry groups, see next section.

Numerical relationships between coding variants and different categories of variable genes

We have established highly specific and constant numerical relationships between the number of PFA-nsSNPs per genome and the number of phase-sensitive genes, and any category of variable genes in general. So the ratio (quotient) of the number of PFA-nsSNPs per genome (median 2,869 in 1,092 genomes, and between 2,655.5 in EAS and 3,672 in AFR in the four ancestry groups) to the total of variable genes defined by presence of ≥ 1 PFA-nsSNPs was 0.71, to the genes with = 1 PFA-nsSNP 0.54, to the phase-sensitive genes with ≥ 2 PFA-nsSNPs 0.17 and their sub-categories with *cis* and *trans* configurations 0.10 and 0.07, respectively (Supplementary Table S6A). Evidently, these ratios, which were calculated from median values, were essentially identical in all ancestry groups and the total set of 1,092 genomes, with minor shifts of 1% in AFR towards phase-sensitive genes and *trans* configurations due to the increased number of PFA-nsSNPs in this ancestry group. Calculating the ratios individually for each of the EUR and AFR genomes, they were found to vary within a very small range; so did the numbers of PFA-nsSNPs per genome and the numbers of variable genes in each of the defined categories. We also refer to these ratios as proportionality constants. Furthermore, also the fractions of PFA-nsSNPs that were found distributed to each of these categories were nearly the same across all ancestry groups (Supplementary Table S6B): about 53–54% of the PFA-nsSNPs per genome generated genes with = 1 PFA-nsSNP and over 46–47% of the PFA-nsSNPs were contained in phase-sensitive genes with ≥ 2 PFA-nsSNPs; of those, 25–26% were found to reside on the same chromosomal homologue (in *cis* configurations) and the remaining 20–22% were located on both homologues of a gene (in *trans* configurations). Again, minor shifts towards phase-sensitive genes (~1–2%) and *trans* configurations (~3–4%) were observed in AFR. Overall, the number of PFA-nsSNPs per phase-sensitive gene remained constant at ~2.7.

Highly specific and constant numerical relationships between the numbers of coding variants per genome and the different categories of variable genes were also obtained when the entirety of nsSNPs were examined, although with different ratios, as shown in Supplementary Table S6C. Analyzing these ratios again individually across the genomes in EUR and AFR, the individual values were within very narrow corridors, as were the numbers of nsSNPs per genome and the numbers of variable genes in each of the defined categories. In this case, the 2.25-fold (AFR 2.2-fold) higher number of nsSNPs per genome resulted in a disproportionately, 2.7-fold (AFR 2.6-fold) higher number of phase-sensitive genes. Thus, these contained a much higher fraction of nsSNPs, 62% (AFR 64%), with 31–33% (AFR 29%) of the nsSNPs residing in *cis* and 29–31% (AFR 35%) in *trans*, the number of nsSNPs per gene again remaining again constant, 3.2 (Supplementary Table S6D). Thus, these results indicate that the considerably higher load of coding variants in this case leads to a disproportionately higher increase of phase-sensitive genes, which accumulate a relatively higher number of variants per gene.

Global sets of variable and phase-sensitive genes: overrepresentation of pathways and GO terms

In this work, we have focused on genes with ≥ 2 PFA-nsSNPs to examine the distribution of variants between the two homologues and global patterns of phase in the diploid human genome. From a biological point of view, however, two different forms of a protein preserving functional flexibility can be generated by any variable gene with at least one PFA-nsSNP encoding a ‘molecular diplotype’ (3). Thus, we examined to which extent the over-representation of described functional content is restricted to phase-sensitive genes, or part of a bigger picture. To this end, we extracted analogously a larger global set of 7,524 genes with ≥ 1 PFA-nsSNPs, which was found significantly enriched for 138 pathways ($P<3.5 \times 10^{-45}$–9.7×10^{-3}) and 177 GO terms ($P<1.09 \times 10^{-37}$–9.6×10^{-4}) (Supplementary Table S10A and B). This set of genes includes the 2,402 phase-sensitive genes that constitute the global set. Subsequently, we determined the relative proportions of the genes with = 1 PFA-nsSNP and ≥ 2 PFA-nsSNPs, respectively, for each of these pathways and GO terms (separately for ‘biological process’, ‘cellular component’ and ‘molecular function’; Supplementary Figure S5A and B). Evidently, the phase-sensitive genes, which represent ~32% of the total set of variable genes, represent a disproportionately large fraction within each pathway and GO term, for instance between 71.4% and 32% in most pathways, supporting their high functional load.

Analysis of *cis*- and *trans*-abundant genes in 1000G ancestry groups and PGP sample

The initial classification of autosomal protein-coding genes has been derived from the global set of 2,402 phase-sensitive genes, which effectively represents 46–72% of these genes within each ancestry group. To test whether this classification is also valid in a population as a whole, we examined each of the ancestry groups separately. Thus, 1,173 significantly *cis*- and 670 *trans*-abundant genes were obtained in EUR, 966 and 590 in EAS, 981 and 497 in AMR and 1,265 and 817 in AFR, accounting for 78–88% of all autosomal phase-sensitive genes. Subsequent analysis of the 184 experimentally haplotype-resolved PGP genomes also validated this classification; correspondingly, 83.5% of the phase-sensitive genes were grouped into 778 significantly *cis*- and 436 *trans*-abundant genes. In sum, these results underscore the existence of *cis*- and *trans*-abundant genes as major categories of variable autosomal genes. Evidently, the group of *cis*-abundant genes is always larger than the group of *trans*-abundant genes, with ratios between 1.55:1 and 2:1. Thus, significant global *cis* abundance is the net result of these two groups. *Cis*- and *trans*-abundant genes were found distributed across the autosomes in varying mixtures, leading to autosomal *cis*/*trans* ratios between ~53:47 (chr. 10) and ~69:31 (chr. 14) (Supplementary Table S12; see also following section). Autosomes with disproportionately higher fractions of *trans*-abundant genes include in addition to chr. 10 the chromosomes 6 and 8, autosomes with a higher density of *cis*-abundant genes in addition to chr. 14 the chromosomes 20 and 22. A visual overview of the distribution of *cis*- and *trans*-abundant genes across the autosomes, that is, of the 'phase-sensitive exome', is provided in Supplementary Figure S7A and B.

Autosomal *cis* fractions

Examining *cis* and *trans* configurations of PFA-nsSNPs per autosome, *cis* fractions were between 52.6% and 68.8% (median values), resulting in a median of ~60% across all autosomes (Supplementary Table S12), equivalent to the global *cis* fraction described. Assessing autosomal *cis* fractions separately per ancestry group unveiled certain patterns: nearly half of the autosomes exhibited very high *cis* fractions between 60% and 71.4% in EUR, EAS and AMR, with the corresponding fractions in AFR being roughly 5% to 20% lower; several autosomes exhibited high *cis* fractions in all four ancestry groups, between ~60% and 73.5%; and few autosomes such as chromosome 6 harboring MHC had comparatively higher *trans* fractions in all ancestry groups, that is, lower *cis* fractions between 56% and 48.8%, the latter value (in AFR) being the lowest overall. Thus, global *cis* abundance is the net result of diverging autosomal *cis*/*trans* ratios, which seems to result from different mixtures of *cis*- and *trans*-abundant genes on a number of autosomes.

Quantitative characterization of gene-based *cis* and *trans* fractions

To characterize *cis*- and *trans*-abundant genes in more detail, we examined first the 1,227 *cis*- and 786 *trans*-abundant genes identified in the global set of phase-sensitive genes (1000G) and subsequently the subsets of these genes which were shared with the experimentally phased genomes (PGP). *Cis*-abundant genes had ≥ 2 PFA-nsSNPs in 23% of the 1,092 genomes (1000G) on average (a fraction corresponding well with the spectrum of average minor allele frequencies (MAFs) assessed for *cis* pairs of PFA-nsSNPs described in Results and Supplementary Figure S3A and B). The PFA-nsSNPs resided in *cis* on average in 82% of the configurations, with a maximum absolute *cis* count of 769 out of a total configuration count of 1,016 in *CDK11B* (Supplementary Table S11A). The gene-based *cis* fractions tended to be high, for example, 35% of the *cis*-abundant genes had *cis* fractions ≥ 90% and 57% had *cis* fractions ≥ 80% of total configuration count. Correspondingly, the (cross-validated) *cis*-abundant genes in PGP had ≥ 2 PFA-nsSNPs in 24% of the 184 genomes, with 90% of the configurations on average residing in *cis*. Furthermore, *trans*-abundant genes had ≥ 2 PFA-nsSNPs in 19% of the 1,092 genomes (1000G) on average, of which 80% (on average) resided in *trans,* with a maximum *trans* count of 1,092 in *MAP2K3* and *OR4C3* (Supplementary Table S11B). Specifically, 29% of all *trans*-abundant genes had gene-based *trans* fractions ≥ 90%, and 50% had *trans* fractions ≥ 80%. Correspondingly, in PGP, *trans*-abundant genes had ≥ 2 PFA-nsSNPs in 22% of the 184 genomes on average, of which 86% resided in *trans.* Remarkably 4% and 8% of the genes in the 1,092 genomes, and 27% and 18% of the genes in the 184 PGP genomes, respectively, consisted solely of *cis,* or *trans* configurations.

Cis- and *trans*-abundance: constant characteristics of autosomal genes

To test whether *cis*- and *trans*-abundance could in effect represent a fairly constant characteristic in autosomal protein-coding genes, we proceeded as follows: we identified, in a first step, those phase-sensitive genes, which were shared by the global set (1000G) and PGP (1,627 genes representing 68% of the global set). We determined the configuration types in this overlap separately for 1000G and PGP. Then we intersected the genes which were *cis*-abundant in 1000G with the genes *trans*-abundant in PGP and vice versa, the genes *trans*-abundant in 1000G with those *cis*-abundant in PGP. The identification of overlaps of 72 and 71 genes, respectively, indicated that 8.7% of the *cis*- and 12.9% of the *trans*-abundant genes had changed configuration type in PGP. Thus, overall, *cis*- and *trans*-abundance seems to represent a relatively constant characteristic of autosomal genes, confirming earlier results.

Cis- and *trans*-abundant genes in relation to haploinsufficiency

To test whether *cis*- and *trans*-abundant genes are related to haploinsufficiency, we have interrogated a previous study that generated predictions of haploinsufficiency (HI) for a total of 12,443 genes based on genomic, sequence and network features (4). Of these, 1,573 genes have a prediction $p \geq 0.7$, i.e. a high predicted probability of being haploinsufficient, and 10,645 genes have a prediction $p < 0.7$. We then intersected these data with our major set of *cis*-abundant genes, i.e. the 1,227 genes identified in the global set of phase-sensitive genes which exhibited a significant excess of *cis* configurations in 1092 genomes (1000G) (Supplementary Table S11A). Of these 1,227 *cis*-abundant genes, a total of 652 genes were found to be included in the HI predictions. The resulting contingency table is shown in Supplementary Table S14A. Thus, the observed number of 38 *cis*-abundant genes that have a HI prediction $p \geq 0.7$ is much smaller than the expected number of genes (n=~84) and therefore highly significant (Chi-squared test with Yates' continuity correction: $p<1.0x10^{-6}$). The same was true for the *trans*-abundant genes. Of the 786 *trans*-abundant genes extracted analogously from 1000G (Supplementary Table S11B), HI predictions were available for 477 genes. Of these, 31 genes have a prediction of $p \geq 0.7$ and 446 genes a prediction of $p < 0.7$; the resulting contingency table is shown in Supplementary Table S14B. The result, again, was highly significant, with observed 31 *trans*-abundant genes that have a HI prediction $p \geq 0.7$ against n=~61 expected genes ($p = 3.03x10^{-5}$, Chi-squared test with Yates' continuity correction). Thus, *cis*- and *trans*-abundance of autosomal genes and HI probability are not independent of each other; *cis*- and *trans*-abundant genes are significantly underrepresented in genes with a high predicted probability of being haploinsufficient.

SUPPLEMENTARY METHODS

Simulation of phased genomes and derivation of expected composite *cis*/*trans* ratios

To corroborate the theoretical assumptions on the composite probability of a *cis* or *trans* configuration as described in Methods, simulations of phase were performed assuming that the variants are distributed randomly between the two homologues of a gene. A virtual set of 1,092 phased genomes was generated as follows: for each virtual genome, random numbers of PFA-nsSNPs were drawn in the range observed in the 1,092 genomes data set (~2,500–3,500) (1). The PFA-nsSNPs were sampled from the total of ~300,000 PFA-nsSNPs annotated in this data set. Phase was simulated assigning to every single PFA-nsSNP in a gene a 50:50 chance to exist on either homologue. Practically this was achieved by randomly drawing with each PFA-nsSNP a phase, i.e. 'homologue 1' or 'homologue 2' attached, from the 1000G database. Accordingly, a random distribution of all nsSNPs between the two homologues was simulated, drawing randomly between ~5,500 and ~7,500 nsSNPs with either homologue 1 or 2 attached from the entire pool of ~1.5 Mio nsSNPs annotated in the 1,092 'real' genomes, generating a second virtual set of 1,092 phased genomes. Two additional virtual sets of 1,092 phased genomes were generated, simulating analogously a random distribution of all sSNPs, and of all nsSNPs and sSNPs combined. To test the validity of our approach to simulate phase, we assessed the *cis*/*trans* ratios separately for 2 up to 5 variants in all virtual data sets, and compared these ratios to the probabilities *P* for these numbers of variants to occur in *cis* under conditions of random distribution, which is $1/2^{n-1}$, with *n* the number of variants. The comparative evaluation showed that the *cis*/*trans* ratios which were generated for defined numbers of variants by simulation were essentially identical to those expected. Thus, the simulated data were considered valid and the (composite) expected *cis*/*trans* ratios across all genomes in the virtual data sets derived. These proved to be in excellent agreement with the theoretically derived composite probability of a *cis*, or *trans* configuration to occur, as described in Methods.

SUPPLEMENTARY DISCUSSION

How could we explain the phenomena described? Preliminary results pointed in principle to two major mechanisms. Firstly, *cis* abundance, with ~60:40 *cis*/*trans* ratios being observed for all types of coding variants, could arise from ancestral admixture as the common underlying mechanism. Further investigations tracing human evolutionary history and population genetic processes will be required to elucidate the apparently ancient origins of *cis* abundance and the specific admixture processes generating it. Moreover, the observation that all populations showed approximately the same *cis*/*trans* ratios (with some decay of LD in AFR leading to a ~55:45 ratio) suggests that genetic admixture between two ancient populations must have occurred before dispersal of modern humans out of Africa and their population expansion worldwide (5,6). Secondly, a significant overrepresentation of particular functional categories in the global sets of phase-sensitive, and *cis*- and *trans*-abundant, genes was observed. With ≥ 2 PFA-nsSNPs, these genes featured an increased mutational load. As preliminary results indicated, processes of ancient balancing selection may have contributed to their higher genetic diversity to preserve their functional flexibility as an adaptive advantage (3,7-9). The observation that all populations had the same distributional patterns of PFA-nsSNPs and functional enrichment suggests that these selective processes must have occurred before ancestral admixture. Thus, this conserved, potentially functionally important ‘phase-sensitive part’ of the diploid human genome may have very ancient origins. This applies particularly also to the common pairs of co-occurring PFA-nsSNPs characterizing *cis*-abundant genes. These could serve as ‘evolutionary signals’ that could contribute to further elucidate the evolutionary history of the phenomena described. In sum, processes of ancient selection followed by admixture may have shaped the overall picture observed.

SUPPLEMENTARY FIGURES

A

Scheme 1. *Cis* and *trans* configurations of variants

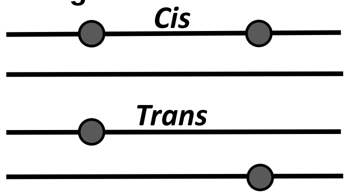

Where diploid autosomal genes have ≥ 2 heterozygous variants, these can either reside on the same chromosomal homologue, in a *cis* configuration, or on both homologues, in a *trans* configuration (Scheme 1). 'Phase' is always determined for nucleotides/alleles different from the reference sequence, also defined as 'non-reference alleles'. The non-reference allele is in the vast majority but not all cases the minor allele. Genes with ≥ 2 heterozygous variants which could exist in either phase configuration are defined as 'phase-sensitive'. The distinction of *cis* and *trans* configurations is based on haplotype information and precisely refers to the *pair* of haplotypes corresponding to the two parental homologues of a specific autosomal gene (primary transcript) in an individual genome.

The pairs of haplotypes under investigation corresponding to the protein-coding regions of the genes are confined functional units where differences in the phase of non-synonymous SNPs (nsSNPs) may have functional consequences. For example, nsSNPs that reside in *cis* leave a second form of the gene intact, while nsSNPs in *trans* may affect both homologous forms of the gene. Phase is most likely to impact gene function and phenotype within genes that contain nsSNPs of potential functional significance. The annotation of nsSNPs is achieved by use of algorithms, for instance by (a combination of) PolyPhen-2 (11), SIFT (12) and GERP (13), which predict whether missense changes caused by nsSNPs alter protein function.

The '*cis*/*trans* ratio' of an individual genome is determined as follows: each autosomal protein-coding gene with ≥ 2 predicted protein function-altering nsSNPs (PFA-nsSNPs) is assigned a *cis* or *trans* configuration. This allows immediate calculation of the *cis* fraction (%) of an individual genome as the number of autosomal genes with *cis* configurations divided by the total number of genes with ≥ 2 PFA-nsSNPs, i.e. total configuration count (equivalent to 100%), and of the *trans* fraction (%) per genome as 100%–*cis* (%). Thus, the *cis*/*trans* ratio of an individual genome represents the ratio of *cis* fraction to *trans* fraction.

As outlined above, the evaluation of *cis* and *trans* configurations is based on a *pair* of haplotypes that correspond to a diploid gene within an individual haplotype-resolved genome. Current approaches do not allow, however, distinction of maternal and paternal haplotypes, but solely the distinction of two different combinations of heterozygous variants, designated 'Haplotype 1' and 'Haplotype 2'.

B

Scheme 2. Expected distribution of *cis*- and *trans* configurations of variants in a population

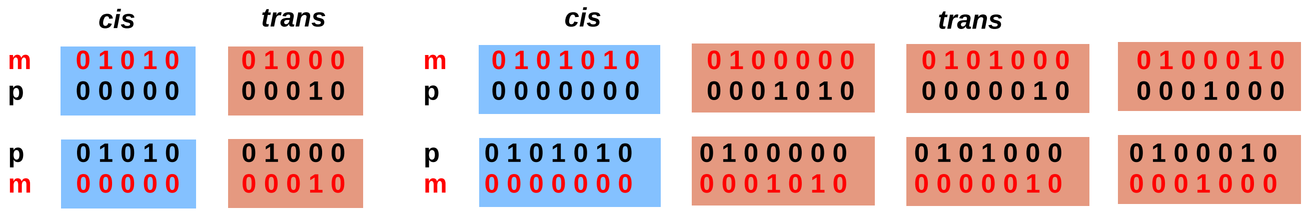

'0', 'reference allele', i.e. allele identical with the reference sequence; '1', 'non-reference allele', i.e. allele different from the reference sequence; m, maternal, p, paternal; blue background *cis* configurations, red background *trans* configurations of variants.

Scheme 2 provides an overview of all different pairs of maternal and paternal haplotypes expected to occur in a population, if a defined number of heterozygous variants (2 on the left, 3 on the right) in a protein-coding gene are distributed randomly between the maternal (m) and paternal (p) homologues. The number of different configurations for a defined number of n variants is 2^n; as illustrated in Scheme 2, for n=2 variants, 4 different configurations would be expected, and for n=3 variants, 8 configurations. Evidently, the same configuration type, i.e. pair of haplotypes, always occurs twice, with the parental origin interchanged. Because current approaches to haplotyping do not allow distinction of maternal and paternal homologues, the maternal and paternal haplotypes are being collapsed into 'Haplotype 1' and 'Haplotype 2'. Thus, in practice, we score a *cis* configuration, or any specific type of a *trans* configuration basically twice, leaving the relative fractions of *cis* and *trans* configurations constant. If the chance for every variant in a gene to occur on either homologue is equal, the expected fraction of *cis* configurations is calculated as $1/2^{n-1}$, n being the number of variants. Obviously (see also Scheme 2), independently of the number of variants, there will always be 2 *cis* configurations, while the fraction of *trans* configurations grows exponentially (Graph).

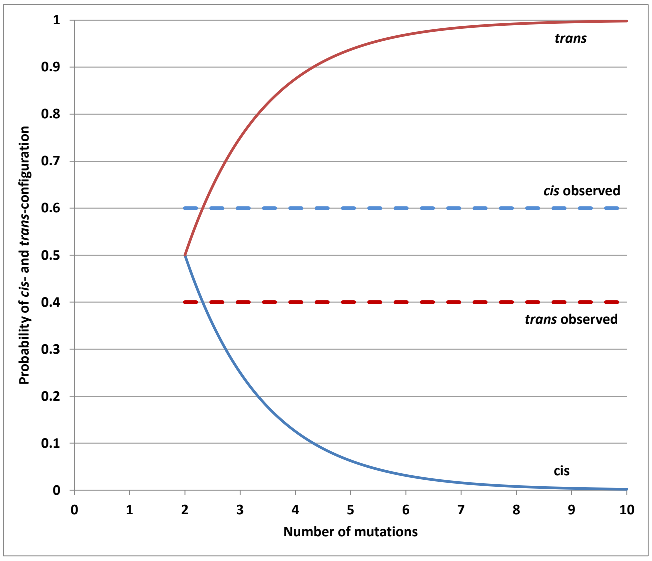

Cis/*Trans* configuration probabilities under random assumptions in relation to the number of variants. *Cis* fraction: blue; *trans* fraction: red. Dashed lines refer to the observed composite *cis*/*trans* ratio of 60:40.

Supplementary Figure S1. *Cis* and *trans* configurations of coding variants in autosomal protein-coding genes. (A) Scheme 1. *Cis* and *trans* configurations of variants. (B) Scheme 2. Expected distribution of *cis*- and *trans* configurations of variants in a population

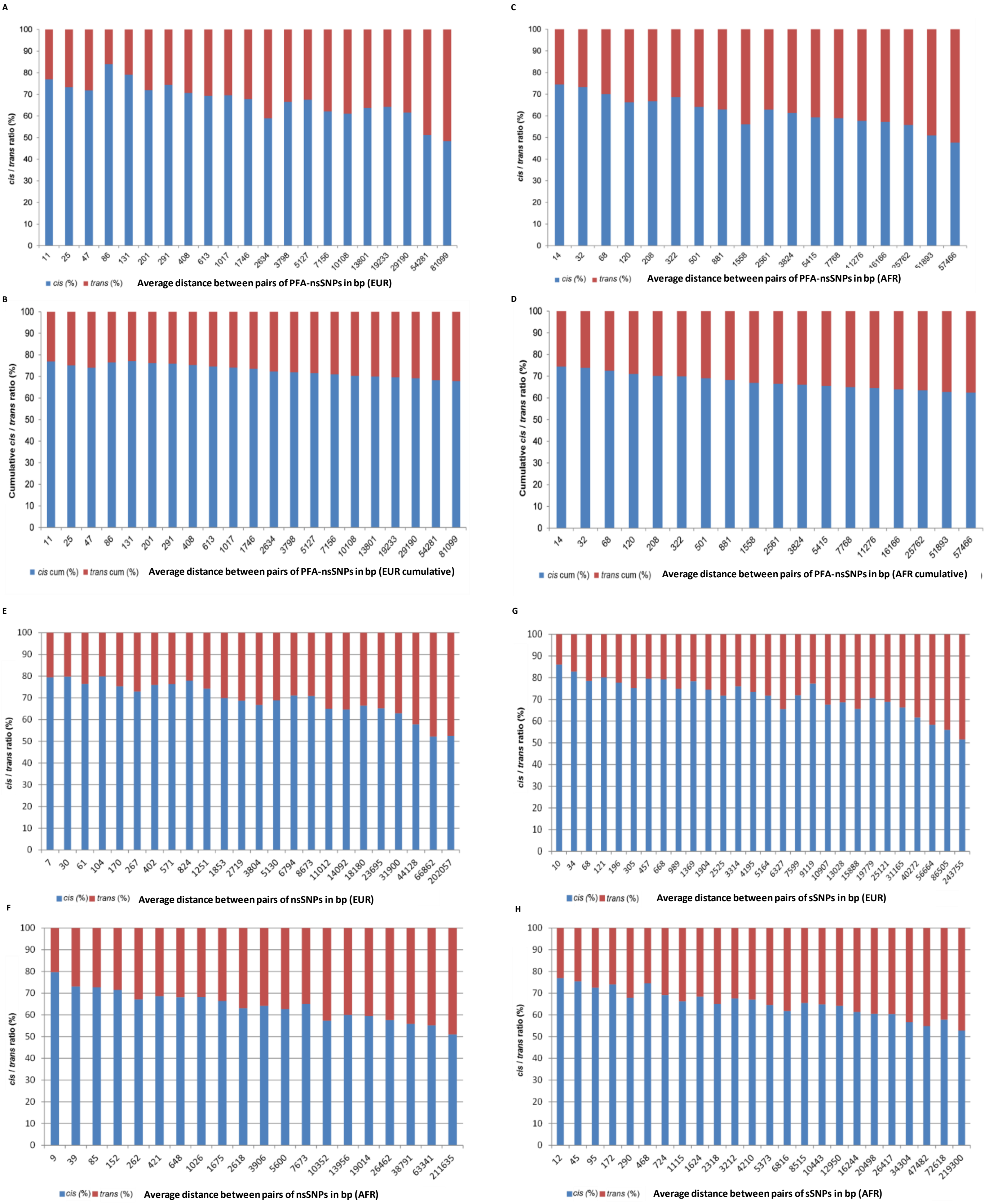

Supplementary Figure S2. Relationship of inter-mutation genome distance with *cis*/*trans* ratio.
(**A**) Relationship of inter-mutation distance with *cis*/*trans* ratio in EUR. *X*-axis**:** Average genome distance (bp) between pairs of predicted protein function-altering nsSNPs (PFA-nsSNPs per bin; the pairs of PFA-nsSNPs were sorted by distance, then binned per 6,000 configurations (i.e. distributed into approximately 20 bins); for each bin, the average genome inter-mutation distance (bp) was calculated and the corresponding *cis*/*trans* ratio assessed; *y*-axis: *cis*/*trans* ratios, with the fraction of *cis* configurations (%) in blue and of *trans* configurations (%) in red. (**B**) Cumulative *cis*/*trans* ratios. (**C**) Relationship of inter-mutation distance with *cis*/*trans* ratio in AFR, analogous to (**A**). (**D**) Cumulative *cis*/*trans* ratios, analogous to (**B**). (**E**) Relationship of inter-mutation distance with *cis*/*trans* ratio for pairs of nsSNPs in EUR. (**F**) Relationship of inter-mutation distance with *cis*/*trans* ratio for pairs of nsSNPs in AFR. (**G**) Relationship of inter-mutation distance with *cis*/*trans* ratio for pairs of synonymous SNPs (sSNPs) in EUR. (**H**) Relationship of inter-mutation distance with *cis*/*trans* ratio for pairs of sSNPs in AFR.

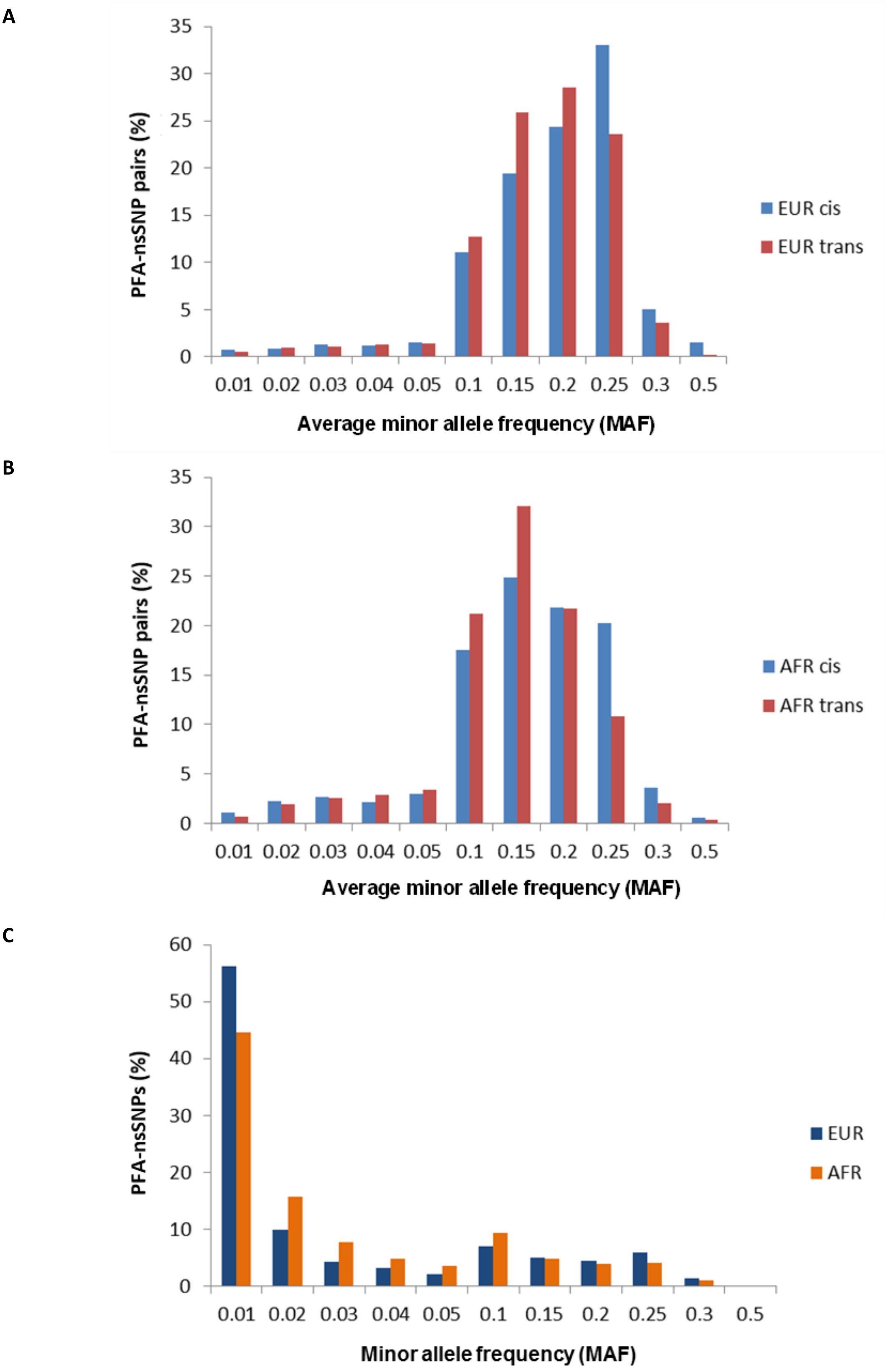

Supplementary Figure S3. Average minor allele frequency (MAF) spectrum of pairs of predicted protein function-altering non-synonymous SNPs (PFA-nsSNPs) in *cis* versus *trans*. (**A**) Average MAF spectrum of pairs of PFA-nsSNPs in *cis* versus *trans* in EUR (1000G); *x*-axis: average MAF per PFA-nsSNP pair, sorted into bins (average MAF calculated from the MAFs of each of the two PFA-nsSNPs as provided by the 1000G database for each ancestry group (1). *Y*-axis: fraction of PFA-nsSNP pairs (%) relative to the total number of pairs of PFA-nsSNPs; blue bars: pairs of PFA-nsSNPs in *cis*; red bars: pairs in *trans*. For instance, 33% of all PFA-nsSNP pairs in *cis* have an average MAF > 0.2 and ≤ 0.25, or, 28% of all PFA-nsSNP pairs in *trans* have an average MAF > 0.15 and ≤ 0.2. (**B**) Analogous to (**A**), average MAF spectrum of pairs of PFA-nsSNPs in *cis* versus *trans* in AFR. (**C**) MAF spectrum of all (single) PFA-nsSNPs contained in all autosomal protein-coding genes (RefSeq) in the 1000G database, for both EUR and AFR. *X*-axis: MAF, binned, presented separately for EUR (orange-brown) and AFR (blue); *y*-axis: fraction of PFA-nsSNPs (%) relative to the total number of PFA-nsSNPs.

A

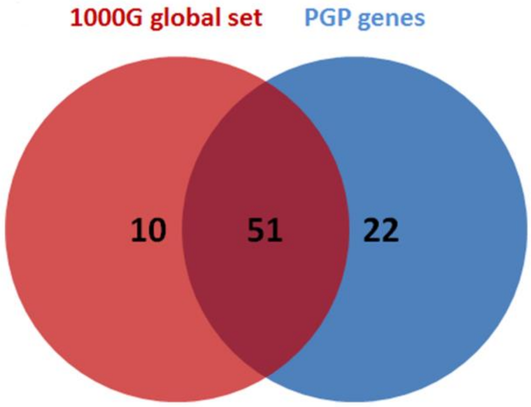

B

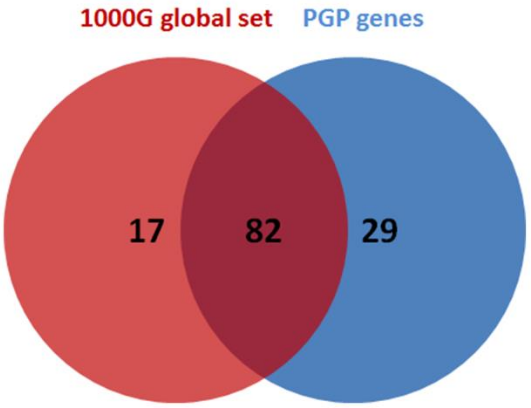

Supplementary Figure S4. Pathways and GO terms shared between 1000G global set and PGP. (**A**) Venn diagram showing the overlap of pathways, which were significantly enriched (*P* < 0.01) in the global set of 2,402 phase-sensitive genes (1000G) (red circle) and the set of 1,627 phase-sensitive genes (*P* < 0.01), which PGP shared with 1000G (blue circle). (**B**) Venn diagram showing the overlap of GO terms, which were significantly enriched (*P* < 0.001) in the global set of 2,402 phase-sensitive genes (1000G) (red circle) and the set of 1,627 phase-sensitive genes (*P* < 0.001), which PGP shared with 1000G (blue circle). The overlap of the sets can be quantified with Sorensen's similarity index, $S = \frac{2ab}{a+b}$, where *a* is the number of genes in the first set, *b* the number of genes in the second set and *ab* the number of genes shared by the two sets. This results in $S = 0.76$ for the similarity between pathways (~76%) and $S = 0.78$ for the similarity between GO terms (~78%).

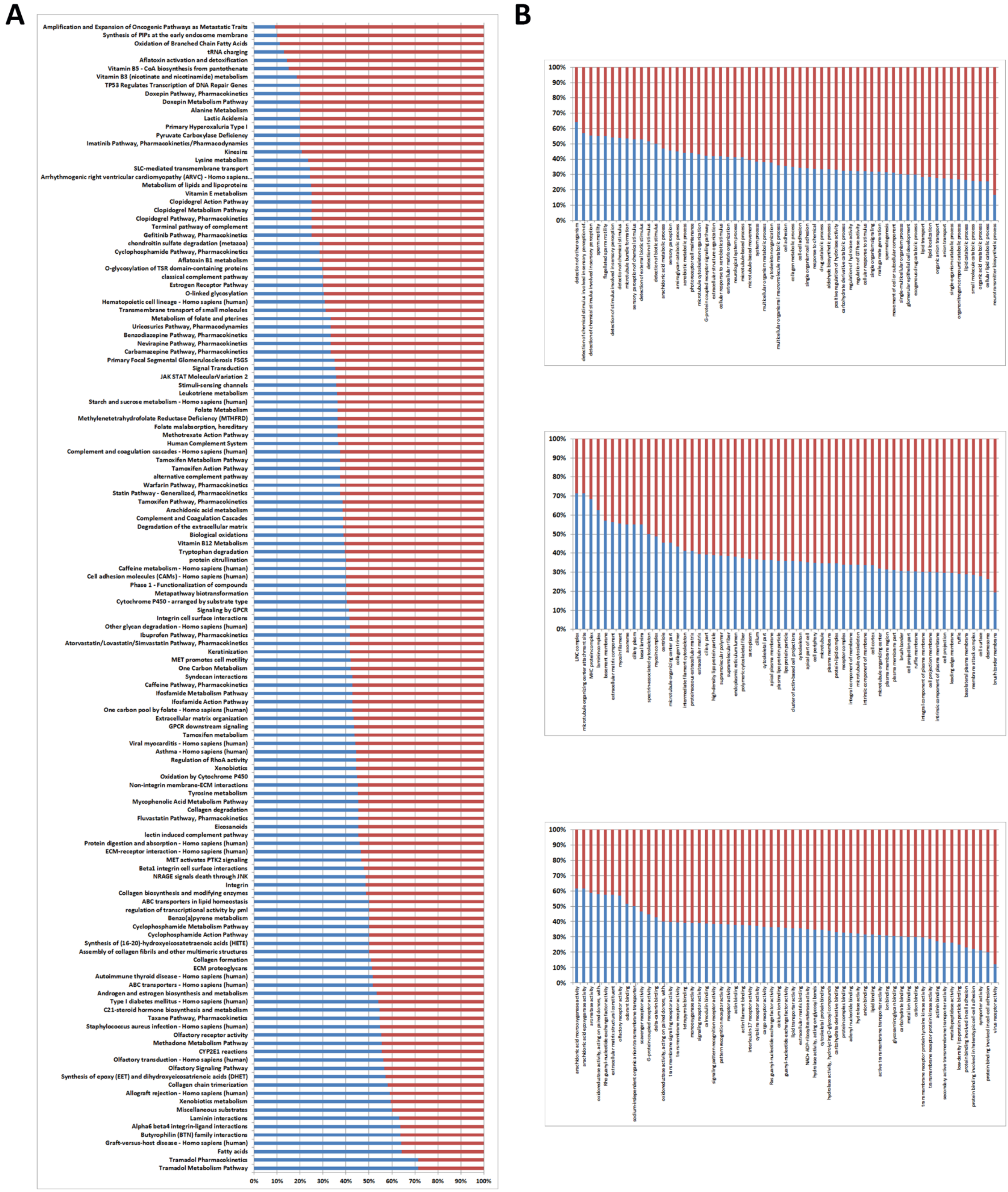

Supplementary Figure S5. Enrichment of global sets of phase-sensitive and variable genes with pathways and GO terms. (**A**) Enrichment of the global set of 7,524 variable genes (which have ≥ 1 protein function-altering nsSNPs (PFA-nsSNPs) in at least one genome in each of the four ancestry groups in 1000G) with pathways. The 138 most significantly enriched pathways ($P < 0.01$) are shown. Blue bars: proportion (%) of the subset of genes with ≥ 2 PFA-nsSNPs, i.e. the genes from the global set of 2,402 phase-sensitive genes (1000G); red bars: proportion (%) of the genes with one PFA-nsSNP. (**B**) Enrichment of the global set of 7,524 variable genes (1000G) with GO terms. The 177 most significantly enriched GO terms ($P < 0.001$) are shown. Blue bars: proportion (%) of the subset of genes with ≥ 2 PFA-nsSNPs, i.e. the global set of phase-sensitive genes (1000G); red bars: proportion (%) of the genes with one PFA-nsSNP. Top: GO terms related to ‘biological process’; middle: GO terms related to ‘cellular component’; bottom: GO terms related to ‘molecular function’.

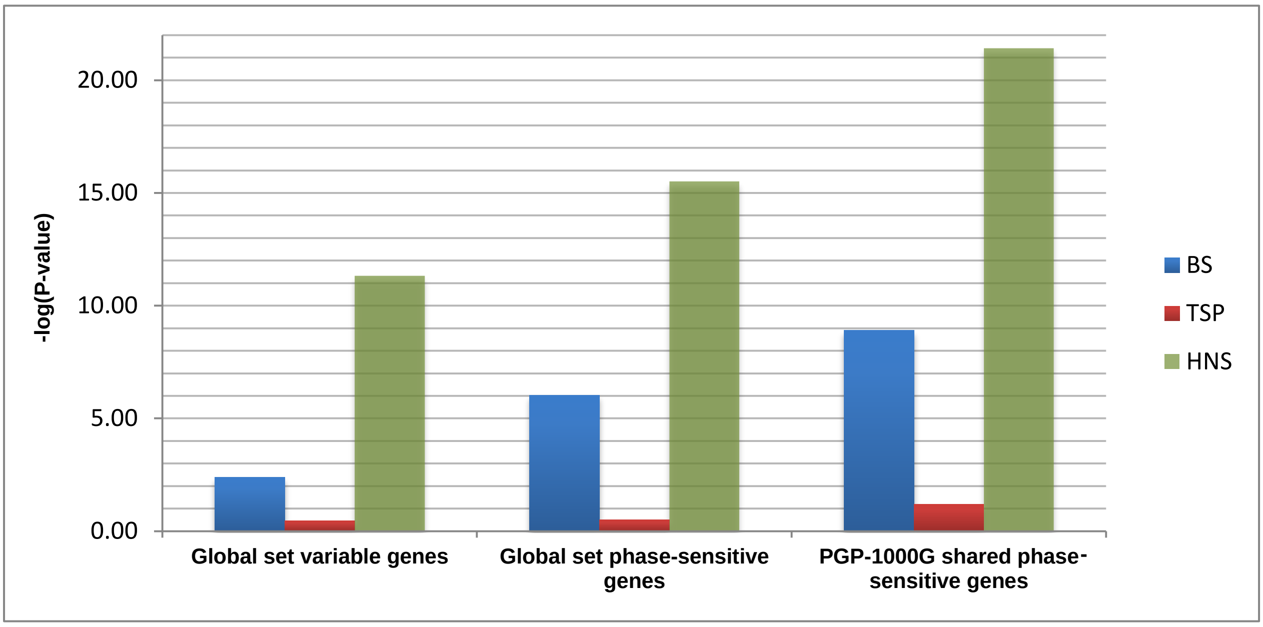

Supplementary Figure S6. Enrichment of variable and phase-sensitive genes with gene sets of evolutionary significance. Panel, left: Blue bar indicates significance of enrichment of the global set of variable genes (1000G), i.e. genes with ≥ 1 predicted protein function-altering nsSNPs (PFA-nsSNPs), with a set of 226 genes reported to evolve under balancing selection (BS); red bar significance of enrichment with a set of 60 genes with any evidence of human-chimpanzee trans-species polymorphisms or haplotypes (TSPs), and green bar enrichment with a set of 104 genes harboring at least one ancient protein-coding SNP or haplotype shared between humans and Neanderthals (HNS); *y*-axis: negative log of the enrichment *P*-value computed with Fisher's exact test. Panel, center: significance of enrichment of the global set of phase-sensitive genes (1000G), i.e. genes with ≥ 2 PFA-nsSNPs, with these gene sets. Panel, right: significance of enrichment of the 1,627 (cross-validated) phase-sensitive genes shared by PGP and the global set of phase-sensitive genes (1000G). The gene sets of potential evolutionary significance are described by Savova et al., 2016 (7).

A

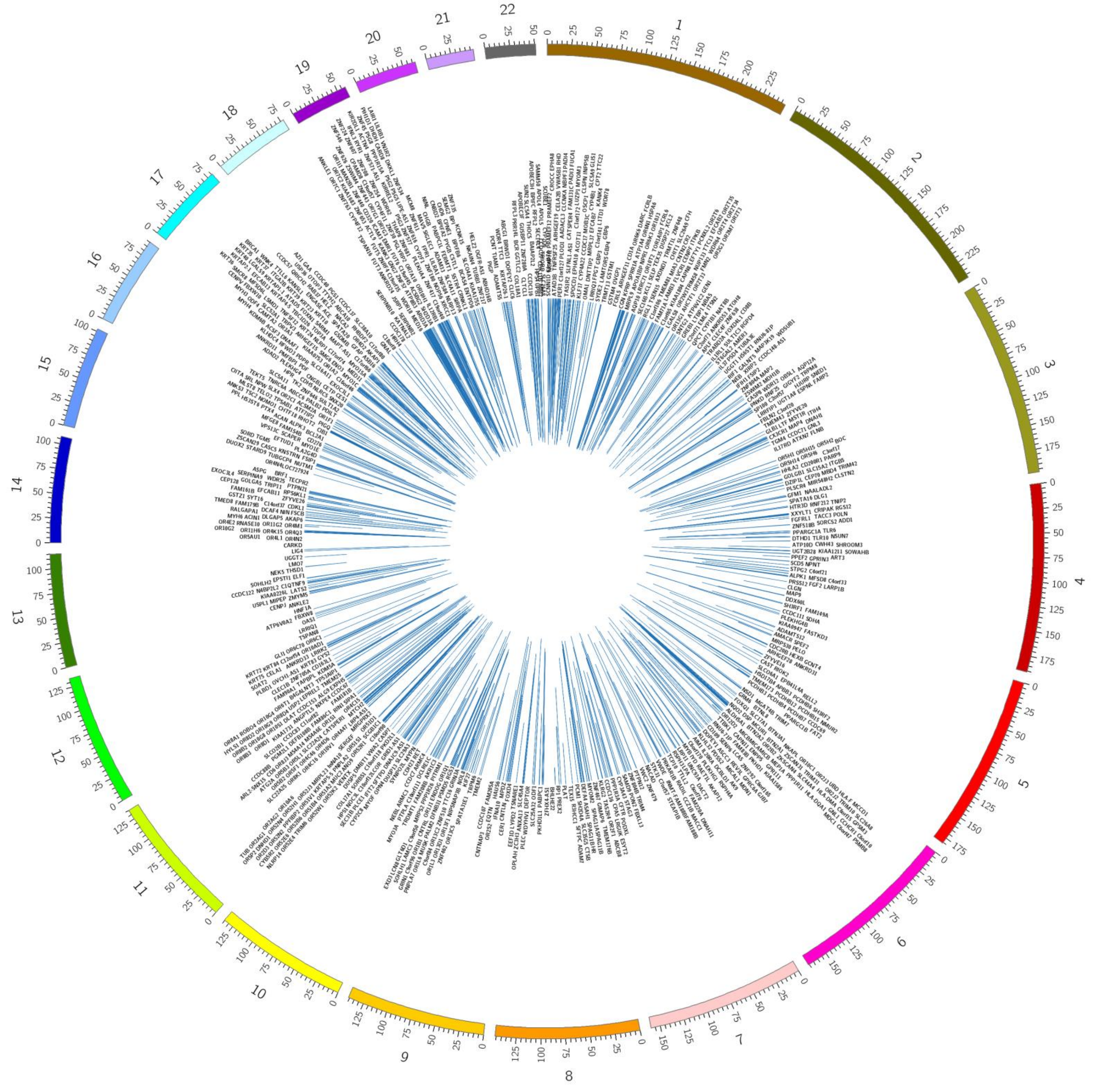

Supplementary Figure S7. Distribution of *cis*- and *trans*-abundant genes across the autosomes.
(**A**) Circos plot describing the distribution of *cis*-abundant genes. In the outer circle, the 22 autosomes are shown, and their assigned respective gene names in the inner circle.

B

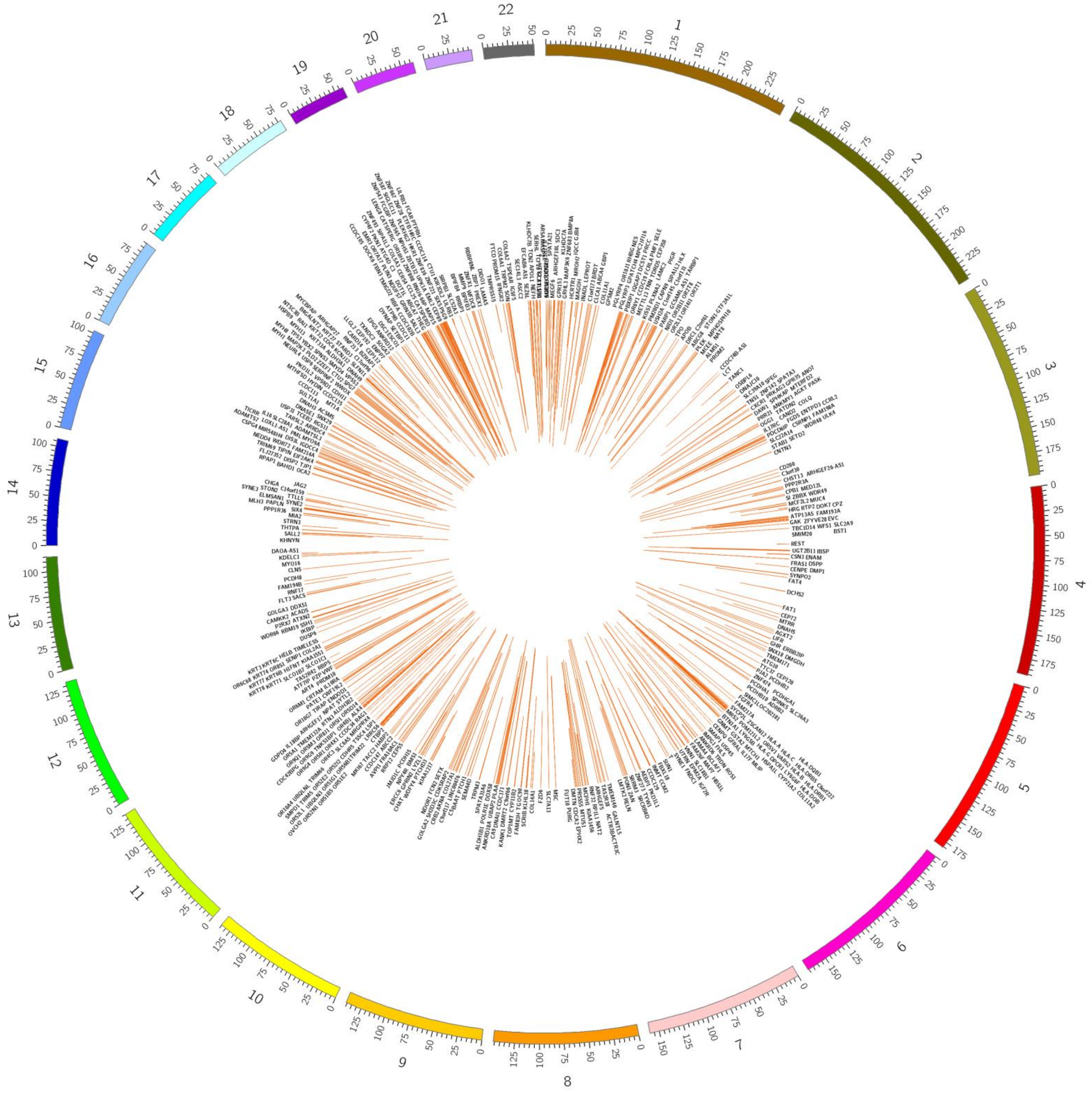

Supplementary Figure S7. Distribution of *cis*- and *trans*-abundant genes across the autosomes.
(**B**) Circos plot describing the distribution of *trans*-abundant genes. In the outer circle, the 22 autosomes are shown, and their assigned respective gene names in the inner circle.

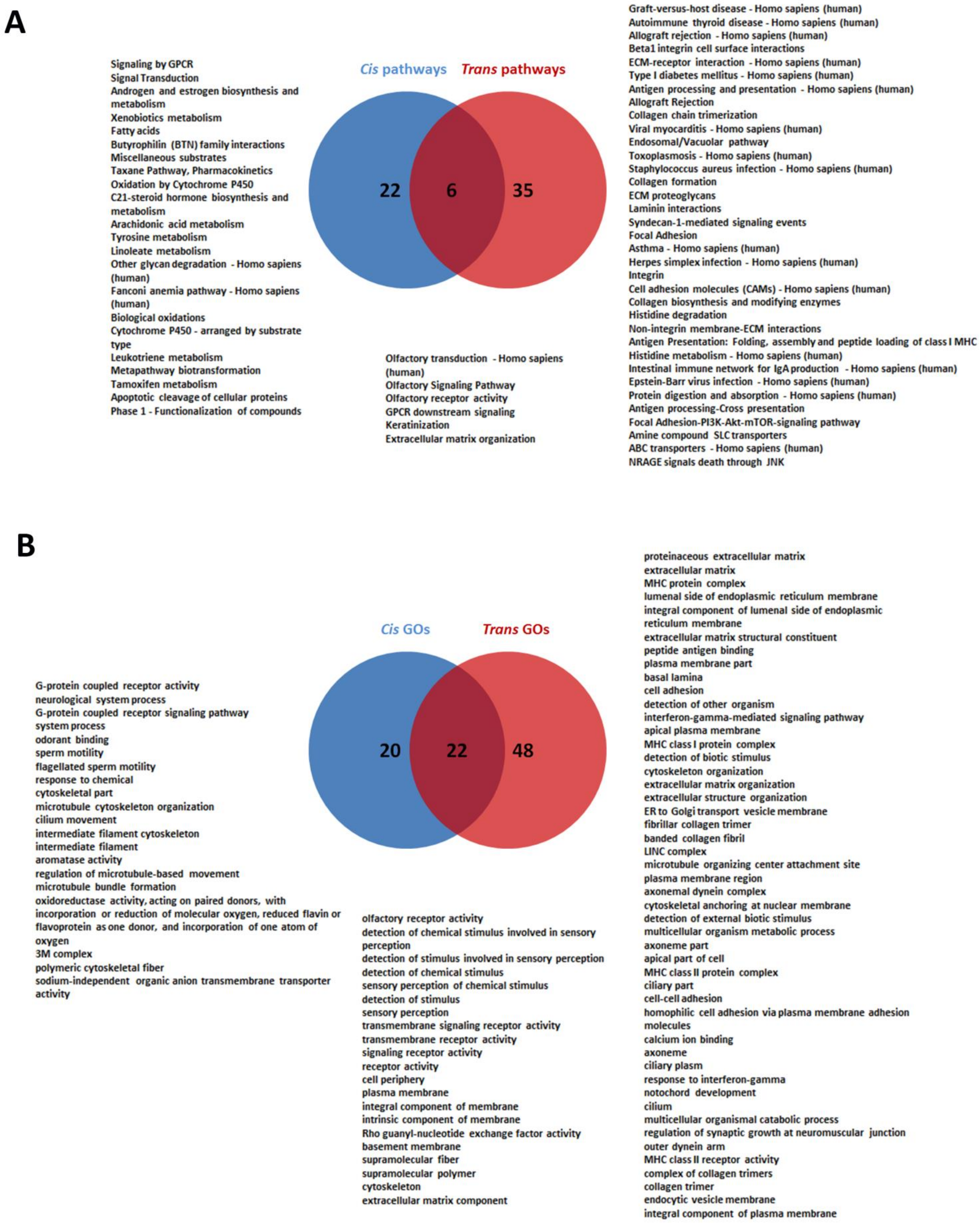

Supplementary Figure S8. Over-representation of pathways and GO terms in *cis*- and *trans*-abundant genes. (**A**) Venn diagram showing the relations between the pathways which are over-represented (P < 0.01) in either *cis*- (left) or *trans*-abundant genes (right); pathways listed in-between, the numbers of which are indicated in the overlap, are enriched in both gene categories; (**B**) Venn diagram illustrating the differential enrichment of GO terms (P < 0.001) in *cis*- (left) and *trans*-abundant genes (right); GO terms listed in-between are enriched in both gene categories.

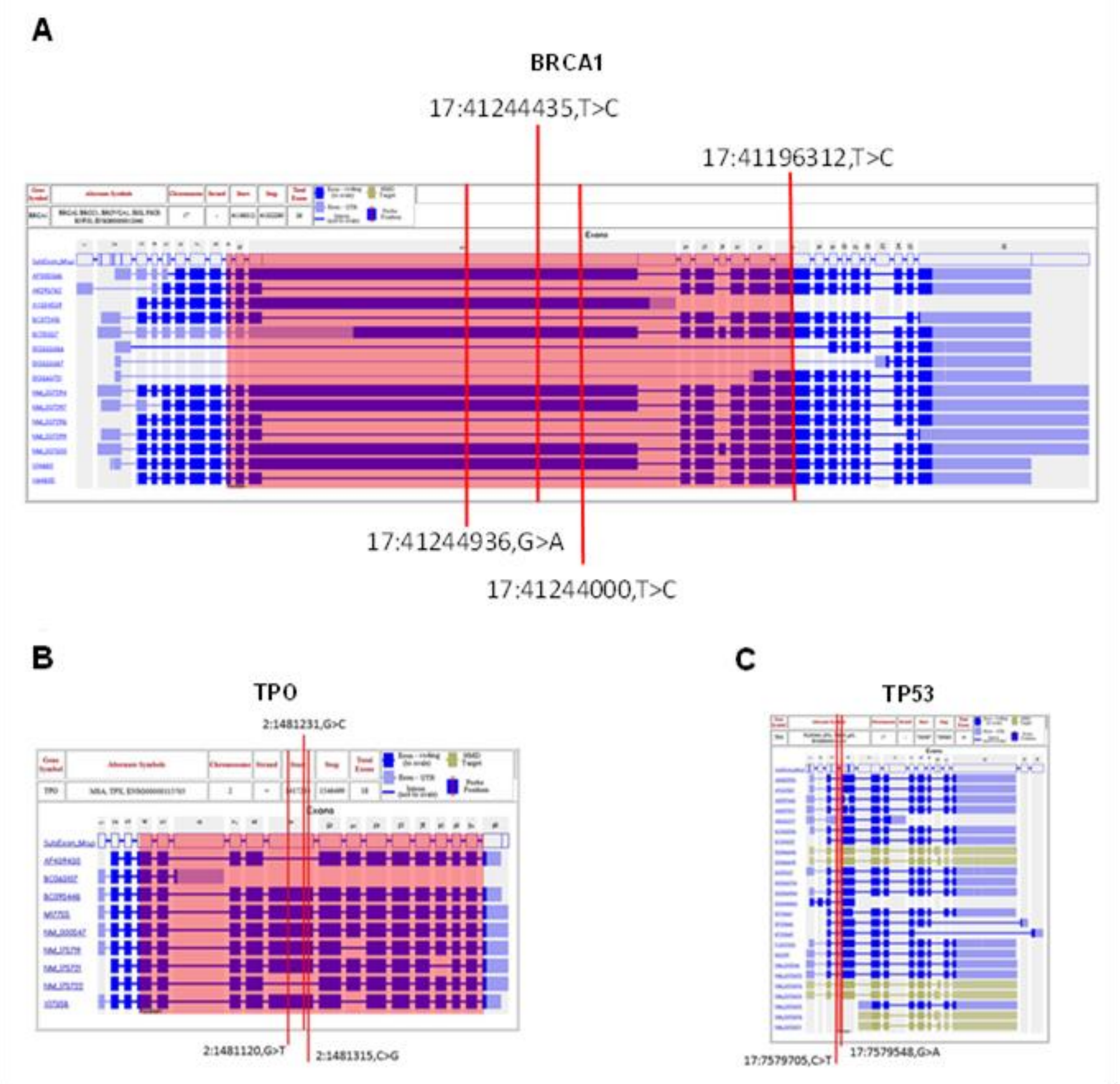

Supplementary Figure S9. Relation between predicted protein function-altering nsSNPs (PFA-nsSNPs) and splicing. Splice variant visualization was carried out with SpliceMiner (10) and PFA-nsSNPs that occur in *cis-* or *trans*-abundant genes have been mapped onto their different splice variants. The genomic region marked with red highlights the occurrence of variants that are within the different *cis* or *trans* configurations. (**A**) *BRCA1* (BReast CAncer 1, early-onset) has *cis* configurations of PFA-nsSNPs in 400 out of the 1092 genomes and *trans* configurations in 63 genomes, thus being highly *cis*-abundant (gene-based *cis* ratio 86%). Overall, the *cis* configurations are combinations of a total of 21 different PFA-nsSNPs in the population spanning the genomic region from exon 9 to exon 17, marked with red. Highlighted are the four most common PFA-nsSNPs. Three of the PFA-nsSNPs are located in the long exon 11 which is frequently spliced, for example between isoforms NM007298 and NM007300. (**B**) *TPO* (Thyroid Peroxidase) has *trans* configurations of PFA-nsSNPs in 626 out of the 1092 genomes and *cis* configurations in 99 genomes, thus being highly trans-abundant (gene-based *trans* ratio 86%). Altogether, the *trans* configurations represent different combinations of a total of 17 different PFA-nsSNPs in the population spanning the genomic region from exon 4 to exon 18, marked with red. The figure highlights three PFA-nsSNPs located in exon 9 which is under splicing, for example in isoform NM175722. (**C**) *TP53* (Tumor Protein P53) has *trans* configurations of PFA-nsSNPs in 255 genomes and *cis* configurations in only 2 genomes, thus being highly *trans*-abundant (gene-based *trans* ratio 99%). The *trans* configurations are composed of the same two PFA-nsSNPs spanning exon 3 to exon 4, marked with red. Both exons are frequently spliced out, for example in isoforms NM001126115, NM001126116 and NM001126117.

SUPPLEMENTARY TABLES

Supplementary Table S1. *Cis* and *trans* configurations of coding variants in 14 populations

Ancestry group[1]	Population[1]	No. phased genomes	*Cis* configs PFA-nsSNPs[2] (%)[5]	*Trans* configs PFA-nsSNPs[2] (%)[6]	*Cis* configs nsSNPs[3] (%)[5]	*Trans* configs nsSNPs[3] (%)[6]	*Cis* configs sSNPs[4] (%)[5]	*Trans* configs sSNPs[4] (%)[6]
EUR	GBR	89	61.3	38.7	60.6	39.4	63.0	37.0
	FIN	93	61.4	38.6	60.8	39.2	62.9	37.1
	IBS	14	61.6	38.4	60.9	39.1	63.1	36.9
	CEU	85	61.6	38.4	61.0	39.0	62.9	37.1
	TSI	98	60.8	39.2	60.4	39.6	62.7	37.3
EAS	CHS	100	59.6	40.4	60.2	39.8	62.8	37.2
	CHB	97	59.5	40.5	60.5	39.5	62.7	37.3
	JPT	89	59.6	40.4	59.8	40.2	63.0	37.0
AMR	PUR	55	60.1	39.9	59.7	40.3	62.3	37.7
	CLM	60	60.1	39.9	59.4	40.6	62.0	38.0
	MXL	66	60.0	40.0	59.6	40.4	62.7	37.3
AFR	YRI	88	54.4	45.6	53.2	46.8	54.7	45.3
	LWK	97	54.0	46.0	53.0	47.0	54.6	45.4
	ASW	61	56.3	43.7	55.6	44.4	56.8	43.2

Results are from statistically haplotype-resolved genomes from the 1000 Genomes (1000G) Project.
[1] Description of ancestry groups and populations in Abecasis et al., 2012 (1).
[2] Predicted protein function-altering non-synonymous SNPs (PFA-nsSNPs) from 1000G annotation database.
[3] nsSNPs from 1000G database.
[4] Synonymous SNPs (sSNPs) from 1000G database.
[5] Values represent the median of *cis* fractions per genome (%); these were assessed as the number of *cis* configurations observed across all autosomal genes (RefSeq hg19 from UCSC table browser), divided by total configuration count.
[6] Analogous to [5].
Configs, configurations.

Supplementary Table S2. *Cis* and *trans* configurations in relation to number of PFA-nsSNPs, nsSNPs and sSNPs in a gene

(**A**) *Cis* and *trans* configurations in relation to number of PFA-nsSNPs

No. PFA-nsSNPs[1] per gene	Sample[2] No. genomes	Total configs[3]		*Cis* configs[4]		*Trans* configs[4]		*Cis*/*Trans* ratio	
		No.	%	No.	%	No.	%	*Cis* (%)[5]	*Trans* (%)[5]
2	1000G 1,092	375,456	66.7	247,220	74.6	128,236	55.4	65.8	34.2
3		104,687	18.6	52,316	15.8	52,371	22.6	50.0	50.0
4		40,826	7.3	19,033	5.7	21,793	9.4	46.6	53.4
5		17,433	3.1	6,919	2.1	10,514	4.5	39.7	60.3
…		…	…	…	…	…	…	…	…
Total		562,698	100	331,250	100	231,448	100	58.9	41.1
2	EUR 379	123,119	67.1	83,432	74.3	39,687	55.8	67.8	32.2
3		33,595	18.3	17,622	15.7	15,973	22.4	52.5	47.5
4		13,366	7.3	6,818	6.1	6,548	9.2	51.0	49.0
5		5,612	3.1	2,384	2.1	3,228	4.5	42.5	57.5
…		…	…	…	…	…	…	…	…
Total		183,536	100	112,362	100	71,174	100	61.2	38.8
2	EAS 286	86,830	67.7	57,947	75.8	28,883	55.8	66.7	33.3
3		22,432	17.5	11,163	14.6	11,269	21.8	49.8	50.2
4		9,005	7.0	4,201	5.5	4,804	9.3	46.7	53.3
5		4,092	3.2	1,865	2.4	2,227	4.3	45.6	54.4
…		…	…	…	…	…	…	…	…
Total		128,212	100	76,436	100	51,776	100	59.6	40.4
2	AMR 181	61,418	66.8	40,873	74.0	20,545	56.0	66.5	33.5
3		16,977	18.5	8,810	16.0	8,167	22.2	51.9	48.1
4		6,680	7.3	3,407	6.2	3,273	8.9	51.0	49.0
5		2,824	3.1	1,108	2.0	1,716	4.7	39.2	60.8
…		…	…	…	…	…	…	…	…
Total		91,925	100	55,213	100	36,712	100	60.1	39.9
2	AFR 246	104,089	65.5	64,968	74.5	39,121	54.5	62.4	37.6
3		31,683	19.9	14,721	16.9	16,962	23.6	46.5	53.5
4		11,775	7.4	4,607	5.3	7,168	10.0	39.1	60.9
5		4,905	3.1	1,562	1.8	3,343	4.7	31.8	68.2
…		…	…	…	…	…	…	…	…
Total		159,025	100	87,239	100	71,786	100	54.9	45.1
2	PGP 184	45,313	64.8	30,264	71.6	15,049	54.4	66.8	33.2
3		13,886	19.9	7,520	17.8	6,366	23.0	54.2	45.8
4		4,716	6.7	2,018	4.8	2,698	9.8	42.8	57.2
5		2,347	3.4	986	2.3	1,361	4.9	42.0	58.0
…		…	…	…	…	…	…	…	…
Total		69,936		42,265		27,671		60.4	39.6

[1] Predicted protein function-altering nsSNPs (PFA-nsSNPs) from 1000 Genomes (1000G) annotation database (1), or annotated by PolyPhen-2 (11) and SIFT (12) as well as GERP conservation scores (13) in the PGP genomes. The number '2' of PFA-nsSNPs per gene for instance means that all genes that have precisely 2 PFA-nsSNPs have been pooled and analysed.
[2] Description of 1000G-derived samples including the four different ancestry groups (EUR, EAS, AMR, AFR) in Abecasis et al., 2012 (1); description of experimentally phased PGP samples in Mao et al., 2016 (2).
[3] Total number of configurations assessed from the genes with given number of PFA-nsSNPs; expressed in addition as fractions (%) relative to the total number of configurations counted from *all* genes across specified number of genomes.
[4] Number of *cis* configurations scored for genes with specified number of PFA-nsSNPs; fractions (%) relative to the total of *cis* configurations counted in *all* genes across specified number of genomes. *Trans* configurations accordingly. The numbers of *cis* and *trans* configurations for any given number of PFA-nsSNPs add up to the corresponding total configurations in [3].
[5] *Cis* fraction (%) calculated for genes with given number of PFA-nsSNPs, dividing the corresponding number of *cis* configurations in [4] by the corresponding total of configurations in [3]; *trans* fractions (%) calculated accordingly.
Configs, configurations.

(**B**) *Cis* and *trans* configurations in relation to number of nsSNPs and sSNPs

Type of variant[1]	No. variants per gene[2]	Total configs[3]		No. *cis* configs[4]	No. *trans* configs[4]	*Cis*/*Trans* ratio	
		No.	%			*Cis* (%)[5]	*Trans* (%)[5]
nsSNPs	2	869,855	57.7	590,886	278,969	67.9	32.1
	3	323,655	21.5	171,127	152,528	52.9	47.1
	4	140,027	9.3	63,395	76,632	45.3	54.7
	5	67,527	4.5	27,505	40,022	40.7	59.3
	…	…	…	…	…	…	…
	Total	1,506,764	100	880,549	626,215	58.4	41.6
sSNPs	2	1,037,140	59.6	725,071	312,069	69.9	30.1
	3	381,027	21.9	204,269	176,758	53.6	46.4
	4	156,911	9.0	70,346	86,565	44.8	55.2
	5	72,263	4.2	28,506	43,757	39.4	60.6
	…	…	…	…	…	…	…
	Total	1,740,093	100	1,053,883	686,210	60.6	39.4
nsSNPs and sSNPs combined	2	1,610,775	47.3	1,106,736	504,039	68.7	31.3
	3	777,497	22.8	416,154	361,343	53.5	46.5
	4	406,970	12.0	181,054	225,916	44.5	55.5
	5	224,911	6.6	87,761	137,150	39.0	61.0
	…	…	…	…	…	…	…
	Total	3,403,910	100	1,900,122	1,503,788	55.8/58.2[6]	44.2/41.8[6]

[1] Non-synonymous SNPs (nsSNPs) and synonymous SNPs (sSNPs) from 1000 Genomes (1000G) Project database (1).
[2] The number ‘2’ of any type of coding variants per gene means that all genes that have precisely 2 variants have been pooled and analysed in 1,092 genomes (1000G). Other numbers analogously.
[3] Total number of configurations assessed from the genes with specified number of variants; expressed in addition as fractions (%) relative to the total number of configurations counted from *all* genes across the 1,092 genomes.
[4] Number of *cis,* or *trans* configurations, respectively, scored for genes with specified number of variants. The numbers of *cis* and *trans* configurations for any specified number of variants add up to the corresponding total configurations in [3].
[5] *Cis* fraction (%) calculated for genes with given number of variants, dividing the corresponding number of *cis* configurations in [4] by the corresponding total of configurations in [3]; *trans* fractions (%) calculated accordingly.
[6] (Bottom right) The median values used in main text to be consistent with the other *cis*/*trans* ratios, are presented in addition. All other values are average values.
Configs, configurations.

Supplementary Table S3. Simulation of expected *cis*/*trans* ratios in relation to number of variants

Type of variant	No. variants per gene[1]	Total configs[2] No.	Total configs[2] %	No. *cis* Configs[3]	No. *trans* Configs[3]	*Cis*/*Trans* ratio Cis (%)[4]	*Cis*/*Trans* ratio Trans (%)[4]
PFA-nsSNPs[5]	2	436,960	66.4	217,789	219,171	49.8	50.2
	3	132,308	20.1	33,327	98,981	25.2	74.8
	4	45,285	6.9	5,669	39,616	12.5	87.5
	5	17,931	2.7	1,112	16,819	6.2	93.8
	…	…	…	…	…	…	…
	Total	657,875	100	258,267	399,608	39.3	60.7
nsSNPs	2	951,313	59.0	475,137	476,176	49.9	50.1
	3	355,549	22.0	89,295	266,254	25.1	74.9
	4	144,799	9.0	18,029	126,770	12.5	87.5
	5	65,041	4.0	4,056	60,985	6.2	93.8
	…	…	…	…	…	…	…
	Total	1,612,714	100	588,494	1,024,220	36.5	63.5
sSNPs	2	1,098,368	60.3	548,709	549,659	50.0	50.0
	3	407,820	22.4	101,771	306,049	25.0	75.0
	4	162,939	8.9	20,387	142,552	12.5	87.5
	5	70,648	3.9	4,448	66,200	6.3	93.7
	…	…	…	…	…	…	…
	Total	1,821,931	100	676,751	1,145,181	37.1	62.9
nsSNPs and sSNPs combined	2	1,803,140	50.1	900,986	902,154	50.0	50.0
	3	848,382	23.6	212,572	635,810	25.1	74.9
	4	415,336	11.5	51,629	363,707	12.4	87.6
	5	211,775	5.9	13,293	198,482	6.3	93.7
	…	…	…	…	…	…	…
	Total	3,599,219	100	1,183,593	2,415,626	32.9	67.1

Simulations of phased genomes were performed as described in Supplementary Methods. To simulate *cis*/*trans* ratios which can be expected under conditions of random distribution of variants between the two homologues, each variant was assigned a 50:50 chance to reside on either one. For each virtual genome, random numbers of each class of coding variants in the range observed in the 1,092 genomes data set were generated; see also Supplementary Methods.

[1] All genes with specified number of variants in the virtual sets of 1,092 genomes (one set generated for each class of coding variants) were pooled and analysed.
[2] Total numbers of configurations assessed from the genes with specified number of variants in virtual set of 1,092 genomes; expressed in addition as fractions (%) relative to the total number of configurations counted from *all* genes across these genomes.
[3] Number of *cis,* or *trans* configurations, respectively, scored for genes with specified number of variants in the 1,092 simulated genomes. The numbers of *cis* and *trans* configurations for any specified number of variants add up to the corresponding total configurations in [2].
[4] *Cis* fraction (%) calculated for genes with given number of variants, dividing the corresponding number of *cis* configurations in [3] by the corresponding total of configurations in [2]; *trans* fractions (%) calculated accordingly.
[5] Predicted protein function-altering non-synonymous SNPs (PFA-nsSNP).
Configs, configurations.

Supplementary Table S4. Inter-mutation genome distances in *cis*- versus *trans* configurations

Population samples[1]	No. phased genomes	*Cis* distance PFA-nsSNPs[2] (bp)	*Trans* distance PFA-nsSNPs[2] (bp)	*Cis* distance nsSNPs[3] (bp)	*Trans* distance nsSNPs[3] (bp)	*Cis* distance sSNPs[4] (bp)	*Trans* distance sSNPs[4] (bp)
1000G	1,092	1,607	5,125	2,130	6,275	3,665	7,761
EUR	379	1,570	5,290	2,248	6,532	3,638	7,982
AFR	246	1,830	4,771	2,189	5,546	3,888	7,228
PGP	184	2,584	5,984	2,058	5,920	3,562	8,280

Inter-mutation genome distances (bp) between totals of specified variant pairs in *cis* versus *trans* are presented.

[1] Description of 1000 Genomes (1000G) sample including ancestry groups EUR and AFR in Abecasis et al., 2012 (1); description of PGP sample in Mao et al., 2016 (2).
[2] Genome distance in bp between pairs of predicted protein function-altering non-synonymous SNPs (PFA-nsSNPs) that reside in *cis* configurations; analogously for pairs in *trans* configurations; annotation from 1000G annotation database (1) or annotated by PolyPhen-2 (11) and SIFT (12) as well as GERP conservation scores (13) in the PGP genomes.
[3] Genome distance in bp between pairs of nsSNPs that reside in *cis* configurations; analogously for those in *trans* configurations.
[4] Genome distance in bp between pairs of synonymous SNPs (sSNPs) that reside in *cis* configurations; analogously for those in *trans* configurations.

Supplementary Table S5. Numbers of phase-sensitive genes and their *cis* and *trans* forms

(**A**) Genes with PFA-nsSNPs[1]

Population samples	No. phased genomes	No. phase-sensitive genes[2] per genome[3,4]	Min – Max	No. genes with *cis* configs per genome[3]	Min – Max	No. genes with *trans* configs per genome[3]	Min – Max
1000G	1,092	487	393 – 710	297	221 – 397	193	132 – 342
EUR	379	484	428 – 554	296	258 – 353	187	145 – 228
EAS	286	449	393 – 505	269	221 – 307	180	147 – 223
AMR	181	510	393 – 613	306	224 – 374	201	132 – 287
AFR	246	647	436 – 710	354	275 – 397	292	161 – 342

[1] Predicted protein function-altering non-synonymous SNPs (PFA-nsSNPs) from the 1000 Genomes annotation database (1).
[2] Defined by presence of ≥ 2 PFA-nsSNPs.
[3] Data represent median values.
[4] Numbers are not equivalent to the sum of genes with *cis* and with *trans* configurations, because median values are used.
Configs, configurations.

(**B**) All genes containing nsSNPs[1]

Population samples	No. phased genomes	No. phase-sensitive genes[2] per genome[3,4]	Min – Max	No. genes with *cis* configs per genome[3]	Min – Max	No. genes with *trans* configs per genome[3]	Min – Max
1000G	1,092	1,317	1,025 – 1,858	796	607 – 1,021	521	402 – 885
EUR	379	1,309	1,195 – 1,437	795	734 – 886	514	452 – 594
EAS	286	1,201	1,044 – 1,298	722	610 – 789	476	419 – 560
AMR	181	1,363	1,025 – 1,664	817	607 – 963	551	402 – 756
AFR	246	1,711	1,210 – 1,858	918	765 – 1,021	793	445 – 885

All genes containing nsSNPs have been analysed, while in Supplementary Table S5A results from the subset of genes containing PFA-nsSNPs are presented.

[1] nsSNPs from the 1000 Genomes annotation database (1).
[2] Defined by presence of ≥ 2 nsSNPs.
[3] Data represent median values.
[4] Numbers are not equivalent to the sum of genes with *cis* and with *trans* configurations, because median values are represented.
Configs, configurations.

Supplementary Table S6. From coding variants to genes to phase configurations

(**A**) From PFA-nsSNPs to genes to phase configurations

Population samples	No. phased genomes	(a) No. PFA-nsSNPs[1] per genome	(b) No. genes with ≥1 PFA-nsSNPs per genome	Quot (b)/(a)	(c) No. genes with =1 PFA-nsSNP per genome	Quot (c)/(a)	(d) No. genes with ≥2 PFA-nsSNPs per genome[2]	Quot (d)/(a)	(e) No. genes with *cis* configs per genome	Quot (e)/(a)	(f) No. genes with *trans* configs per genome	Quot (f)/(a)
1000G	1,092	2,869	2,023	0.71	1,541	0.54	487	0.17	297	0.10	193	0.07
EUR	379	2,847	2,015	0.71	1,532	0.54	484	0.17	296	0.10	187	0.07
EAS	286	2,656	1,872	0.71	1,422	0.54	449	0.17	269	0.10	180	0.07
AMR	181	2,956	2,088	0.71	1,576	0.53	510	0.17	306	0.10	201	0.07
AFR	246	3,672	2,557	0.70	1,907	0.52	647	0.18	354	0.10	292	0.08

[1] Predicted protein function-altering non-synonymous SNPs (PFA-nsSNPs) from 1000 Genomes annotation database (1).
[2] Numbers are not equivalent to the sum of genes with *cis* and *trans* configurations, because median values are used. Essentially all data represent median values.
Quot, quotient; configs, configurations.

(**B**) Distribution of PFA-nsSNPs between gene categories

Population samples	No. phased genomes	No. PFA-nsSNPs[1] per genome	PFA-nsSNPs (%)[2] in genes with =1 PFA-nsSNP	PFA-nsSNPs (%)[3] in genes with ≥2 PFA-nsSNPs	No. PFA-nsSNPs per gene with ≥2 PFA-nsSNPs[4]	PFA-nsSNPs (%)[5,7] in genes with *cis* configs	PFA-nsSNPs (%)[6,7] in genes with *trans* configs
1000G	1,092	2,869	53.24	46.76	2.73	24.81	21.95
EUR	379	2,847	53.96	46.04	2.72	25.66	20.38
EAS	286	2,656	53.8	46.2	2.75	24.66	21.54
AMR	181	2,956	53.07	46.93	2.71	25.55	21.38
AFR	246	3,672	52.02	47.98	2.73	23.47	24.51

[1] PFA-nsSNPs from 1000 Genomes annotation database (1).
[2] Number of PFA-nsSNPs contained in this gene category divided by total number of PFA-nsSNPs per genome.
[3] Number of PFA-nsSNPs contained in genes with ≥ 2 PFA-nsSNPs divided by total number of PFA-nsSNPs per genome.
[4] Number of PFA-nsSNPs per phase-sensitive gene, i.e. per gene with ≥2 PFA-nsSNPs (average across all phase-sensitive genes).
[5] Number of PFA-nsSNPs contained in *cis* configurations divided by total number of PFA-nsSNPs per genome.
[6] Number of PFA-nsSNPs contained in *trans* configurations divided by total number of PFA-nsSNPs per genome.
[7] Median values are used.

(**C**) From all nsSNPs to genes to phase configurations

Population samples	No. phased genomes	(a) No. nsSNPs[1] per genome	(b) No. genes with ≥1 nsSNPs per genome	Quot (b)/(a)	(c) No. genes with =1 nsSNP per genome	Quot (c)/(a)[3]	(d) No. genes with ≥2 nsSNPs per genome[2]	Quot (d)/(a)	(e) No. genes with *cis* configs per genome	Quot (e)/(a)	(f) No. genes with *trans* configs per genome	Quot (f)/(a)
1000G	1,092	6,456	3,771	0.58	2,456	0.38	1,317	0.20	796	0.12	521	0.08
EUR	379	6,429	3,759	0.59	2,447	0.38	1,309	0.20	795	0.12	514	0.08
EAS	286	5,972	3,483	0.58	2,283	0.38	1,201	0.20	722	0.12	476	0.08
AMR	181	6,676	3,876	0.58	2,511	0.38	1,363	0.20	817	0.12	551	0.08
AFR	246	8,229	4,689	0.57	2,974	0.36	1,711	0.21	918	0.11	793	0.10

[1] nsSNPs from 1000 Genomes annotation database (1).
[2] Numbers are not equivalent to the sum of genes with *cis* and *trans* configurations, because median values are used. Essentially all data represent median values.
Quot, quotient; configs, configurations.

(**D**) Distribution of nsSNPs between gene categories

Population samples	No. phased genomes	No. nsSNPs[1] per genome	nsSNPs (%)[2] in genes with =1 nsSNPs	nsSNPs (%)[3] in genes with ≥2 nsSNPs	No. nsSNPs per gene with ≥2 nsSNPs[4]	nsSNPs (%)[5,7] in genes with *cis* configs	nsSNPs (%)[6,7] in genes with *trans* configs
1000G	1,092	6,456	37.57	62.43	3.20	31.32	31.11
EUR	379	6,429	38.13	61.87	3.04	32.60	29.27
EAS	286	5,972	38.34	61.66	3.07	31.78	29.88
AMR	181	6,676	37.58	62.42	3.06	32.06	30.36
AFR	246	8,229	36.25	63.75	3.06	28.93	34.82

[1] nsSNPs from 1000 Genomes annotation database (1).
[2] Number of nsSNPs contained in this gene category divided by total number of nsSNPs per genome.
[3] Number of nsSNPs contained in genes with ≥ 2 nsSNPs divided by total number of nsSNPs per genome.
[4] Number of nsSNPs per phase-sensitive gene, i.e. per gene with ≥ 2 nsSNPs (average of nsSNPs across all phase-sensitive genes).
[5] Number of nsSNPs contained in *cis* configurations divided by number of nsSNPs per genome.
[6] Number of nsSNPs contained in *trans* configurations divided by number of nsSNPs per genome.
[7] Median values are used.

Supplementary Table S12. *Cis* configurations of PFA-nsSNPs[1] per autosome

		Cis configurations (%)[2]			
	1,092	EUR	EAS	AMR	AFR
chr1	63.1	65.6	64.3	63.6	55.1
chr2	58.6	59.1	60.8	58.1	56.3
chr3	60.0	61.9	61.1	62.1	53.8
chr4	62.5	64.7	66.7	62.5	52.4
chr5	60.9	65.0	60.0	61.9	55.2
chr6	53.1	56.0	52.6	54.2	48.8
chr7	60.0	62.5	60.0	60.0	55.7
chr8	52.9	53.8	53.8	53.3	49.0
chr9	55.9	59.1	52.5	59.1	52.8
chr10	52.6	54.5	50.0	54.2	52.0
chr11	60.6	60.0	62.2	60.9	60.0
chr12	54.5	57.1	50.0	52.9	54.5
chr13	62.5	66.7	66.7	66.7	57.1
chr14	68.8	71.4	73.5	68.8	61.5
chr15	56.3	55.6	61.5	57.1	50.9
chr16	61.9	66.7	60.0	65.0	55.4
chr17	57.6	59.4	58.5	57.6	55.9
chr18	55.6	50.0	57.1	55.6	55.6
chr19	60.6	61.9	61.1	62.0	58.3
chr20	64.3	68.8	66.7	64.3	56.8
chr21	60.0	66.7	60.0	62.5	50.0
chr22	66.7	71.4	62.5	70.0	50.0
Median	60.0	61.9	60.4	61.4	55.2
Min	52.6	50.0	50.0	52.9	48.8
Max	68.8	71.4	73.5	70.0	61.5

Results are from statistically haplotype-resolved genomes from the 1000 Genomes (1000G) Project.

[1] Predicted protein function-altering non-synonymous SNPs (PFA-nsSNPs)
[2] Values represent the median of *cis* fractions (%)per autosome per genome; these were assessed as the number of *cis* configurations observed across all autosomal protein-coding genes contained in designated autosome, divided by total configuration count per autosome, calculated across the 1092 genomes and each of the ancestry groups.

Supplementary Table S14. *Cis*- and *trans*-abundance and haploinsufficiency

(**A**) Contingency table describing genes which are *cis*-abundant and not *cis*-abundant

	HI $p \geq 0.7$	HI $p<0.7$	Total
***cis*-abundant**	38	614	652
**not *cis*-abundant**	1,535	10,031	11,566
**Total**	1,573	10,645	12,218

HI ($p \geq 0.7$) high predicted probability of being haploinsufficient
HI ($p < 0.7$) lower predicted probability of being haploinsufficient

(B) Contingency table describing genes which are *trans*-abundant and not *trans*-abundant

	HI $p \geq 0.7$	HI $p<0.7$	Total
***trans*-abundant**	31	446	477
**not t*rans*-abundant**	1,542	10,199	11,741
**Total**	1,573	10,645	12,218

HI ($p \geq 0.7$) high predicted probability of being haploinsufficient
HI ($p < 0.7$) lower predicted probability of being haploinsufficient

SUPPLEMENTARY REFERENCES

1. Abecasis, G.R., Auton, A., Brooks, L.D., DePristo, M.A., Durbin, R.M., Handsaker, R.E., Kang, H.M., Marth, G.T. and McVean, G.A. (2012) An integrated map of genetic variation from 1,092 human genomes. *Nature*, **491**, 56-65.
2. Mao, Q., Ciotlos, S., Zhang, R.Y., Ball, M.P., Chin, R., Carnevali, P., Barua, N., Nguyen, S., Agarwal, M.R., Clegg, T. *et al.* (2016) The whole genome sequences and experimentally phased haplotypes of over 100 personal genomes. *Gigascience*, **5**, 42.
3. Hoehe, M.R., Church, G.M., Lehrach, H., Kroslak, T., Palczewski, S., Nowick, K., Schulz, S., Suk, E.K. and Huebsch, T. (2014) Multiple haplotype-resolved genomes reveal population patterns of gene and protein diplotypes. *Nat Commun*, **5**, 5569.
4. Huang, N., Lee, I., Marcotte, E.M. and Hurles, M.E. (2010) Characterising and predicting haploinsufficiency in the human genome. *PLoS Genet*, **6**, e1001154.
5. Nielsen, R., Akey, J.M., Jakobsson, M., Pritchard, J.K., Tishkoff, S. and Willerslev, E. (2017) Tracing the peopling of the world through genomics. *Nature*, **541**, 302-310.
6. McEvoy, B.P., Powell, J.E., Goddard, M.E. and Visscher, P.M. (2011) Human population dispersal "Out of Africa" estimated from linkage disequilibrium and allele frequencies of SNPs. *Genome Res*, **21**, 821-829.
7. Savova, V., Chun, S., Sohail, M., McCole, R.B., Witwicki, R., Gai, L., Lenz, T.L., Wu, C.T., Sunyaev, S.R. and Gimelbrant, A.A. (2016) Genes with monoallelic expression contribute disproportionately to genetic diversity in humans. *Nat Genet*, **48**, 231-237.
8. Wu, C.t. and Dunlap, J.C. (2002) In Jay, C. D. and Wu, C.-t. (eds.), *Advances in Genetics*. Academic Press, Vol. Volume 46, pp. xvii-xxiii.
9. Sellis, D., Callahan, B.J., Petrov, D.A. and Messer, P.W. (2011) Heterozygote advantage as a natural consequence of adaptation in diploids. *Proc Natl Acad Sci U S A*, **108**, 20666-20671.
10. Kahn, A.B., Ryan, M.C., Liu, H., Zeeberg, B.R., Jamison, D.C. and Weinstein, J.N. (2007) SpliceMiner: a high-throughput database implementation of the NCBI Evidence Viewer for microarray splice variant analysis. *BMC Bioinformatics*, **8**, 75.
11. Adzhubei, I.A., Schmidt, S., Peshkin, L., Ramensky, V.E., Gerasimova, A., Bork, P., Kondrashov, A.S. and Sunyaev, S.R. (2010) A method and server for predicting damaging missense mutations. *Nat Methods*, **7**, 248-249.
12. Kumar, P., Henikoff, S. and Ng, P.C. (2009) Predicting the effects of coding non-synonymous variants on protein function using the SIFT algorithm. *Nat Protoc*, **4**, 1073-1081.
13. Cooper, G.M., Stone, E.A., Asimenos, G., Program, N.C.S., Green, E.D., Batzoglou, S. and Sidow, A. (2005) Distribution and intensity of constraint in mammalian genomic sequence. *Genome Res*, **15**, 901-913.
